# Quaternization of Composite Algal/PEI Beads for Enhanced Uranium Sorption—Application to Ore Acidic Leachate

**DOI:** 10.3390/gels6020012

**Published:** 2020-03-30

**Authors:** Mohammed F. Hamza, Amal E. Mubark, Yuezou Wei, Thierry Vincent, Eric Guibal

**Affiliations:** 1 Guangxi Key Laboratory of Processing for Non-ferrous Metals and Featured Materials, School of Resources, Environment and Materials, Guangxi University, Nanning 530004, China; m_fouda21@hotmail.com; 2 Nuclear Materials Authority, POB 530, El-Maadi, Cairo11835, Egypt; amal.mubark@yahoo.com; 3 Department of Nuclear Engineering and Radiological Sciences (NERS), Shanghai Jiao Tong University, Shanghai 200240, China; 4 Polymers Composites and hybrids (PCH), IMT-Mines Ales, 6, avenue de Clavières, F-30319 Alès cedex, France; thierry.vincent@mines-ales.fr

**Keywords:** algal/Polyethylenimine beads, quaternization, uranyl sorption and desorption, sorption isotherms and uptake kinetics, metal recovery from ore leachates

## Abstract

The necessity to recover uranium from dilute solutions (for environmental/safety and resource management) is driving research towards developing new sorbents. This study focuses on the enhancement of U(VI) sorption properties of composite algal/Polyethylenimine beads through the quaternization of the support (by reaction with glycidyltrimethylammonium chloride). The sorbent is fully characterized by FTIR, XPS for confirming the contribution of protonated amine and quaternary ammonium groups on U(VI) binding (with possible contribution of hydroxyl and carboxyl groups, depending on the pH). The sorption properties are investigated in batch with reference to pH effect (optimum value: pH 4), uptake kinetics (equilibrium: 40 min) and sorption isotherms (maximum sorption capacity: 0.86 mmol U g^−1^). Metal desorption (with 0.5 M NaCl/0.5 M HCl) is highly efficient and the sorbent can be reused for five cycles with limited decrease in performance. The sorbent is successfully applied to the selective recovery of U(VI) from acidic leachate of uranium ore, after pre-treatment (cementation of copper, precipitation of rare earth elements with oxalate, and precipitation of iron). A pure yellow cake is obtained after precipitation of the eluate.

## 1. Introduction

The demand of uranium for nuclear applications makes the extraction of this metal a strategic issue in terms of both economic and strategic aspects. In addition, this radionuclide is toxic and its exploitation (mining and processing) may cause significant hazards for the environment and surrounding populations. These different reasons may explain the interest of the research community for developing techniques for processing of ores (hydrometallurgy and leaching), and recovery of uranyl from aqueous solutions. Depending on the geological origin of the uranium-bearing ores, leaching may be processed using alkaline [1] or an acidic route [2]. However, in most cases the metal is extracted by acidic leaching [3,4,5]. The acid way is usually considered non-selective and produces multi-metal solutions such as base metals, rare earth elements (REEs), in addition to uranium. Separation and enrichment (the concentration effect) are thus important pieces in the design of the global ore processing.

The treatment of acidic leachates may involve a wide range of techniques including selective precipitations [6], solvent extraction [7,8,9], and impregnated resins [10,11,12]. However, sorption processes have retained more attention for the treatment of low-concentration effluents. Uranium sorption was reported using biosorbents [13,14,15,16], chelating resins [17,18,19,20], and ion-exchange resins [21,22,23,24,25,26], and functionalized inorganic supports [27,28,29,30,31,32,33,34].

The speciation of metal ions is a critical parameter that controls the affinity of the sorbents for metal species [23,35,36]. Recently, a new generation of porous material (Algal/Polyethyleneimine beads, APEI) was synthesized through the interaction of alginate and brown algae biomass with polyethylenimine (PEI) [37,38,39]. These materials may combine chelation and ion-exchange properties, depending on the pH, and the metal ion (including its speciation). The concept is based on the partial extraction of alginate from brown algae in alkaline conditions, followed by the ionic interaction of carboxylate groups of alginate with protonated amine groups of PEI. This first polymer network is strengthen by the crosslinking of amine groups with glutaraldehyde and the ionotropic gelation of carboxylate groups with calcium. This second network contributes to reinforce the stability of the composite. This original method uses renewable resources (alginate and algal biomass) and environmentally friendly synthetic polymers (PEI). The synthesis procedure is remarkably simple.

However, these materials can be also used as support for functionalization with the target of increasing the reactivity and/or the selectivity of metal sorption. For example, an amidoxime derivative of APEI (AO-APEI) [40] was developed for the sorption of Sr(II) from aqueous solutions, including in seawater. More recently, APEI was quaternized by grafting glycidyltrimethylammonium moieties on the composite beads (Q-APEI). This sorbent was efficiently tested for the sorption of Sc(III), especially for the recovery of the metal from complex effluent issued from the treatment of red mud [41]. The properties of these materials have been fully characterized in terms of composition (elemental analysis, FTIR, and XPS), textural properties and thermogravimetric analysis.

The present study investigates the sorption properties of Q-APEI for the sorption of uranyl ions from acidic solutions. The interactions of the sorbent with uranyl ions are characterized by FTIR and XPS analysis. In a second step, the sorption properties are extensively investigated, including: pH effect, study of uptake kinetics and sorption isotherms at optimum pH, metal desorption, and sorbent recycling. The last part of the work focuses on the treatment of sulfuric acid leachates of polymetallic ore. A series of pre-treatments is used for separating the metals before using Q-APEI for the recovery of uranyl ions as a yellow cake.

## 2. Results and Discussion

### 2.1. Material Characterization

#### 2.1.1. FTIR Analysis

**Figure 1:**
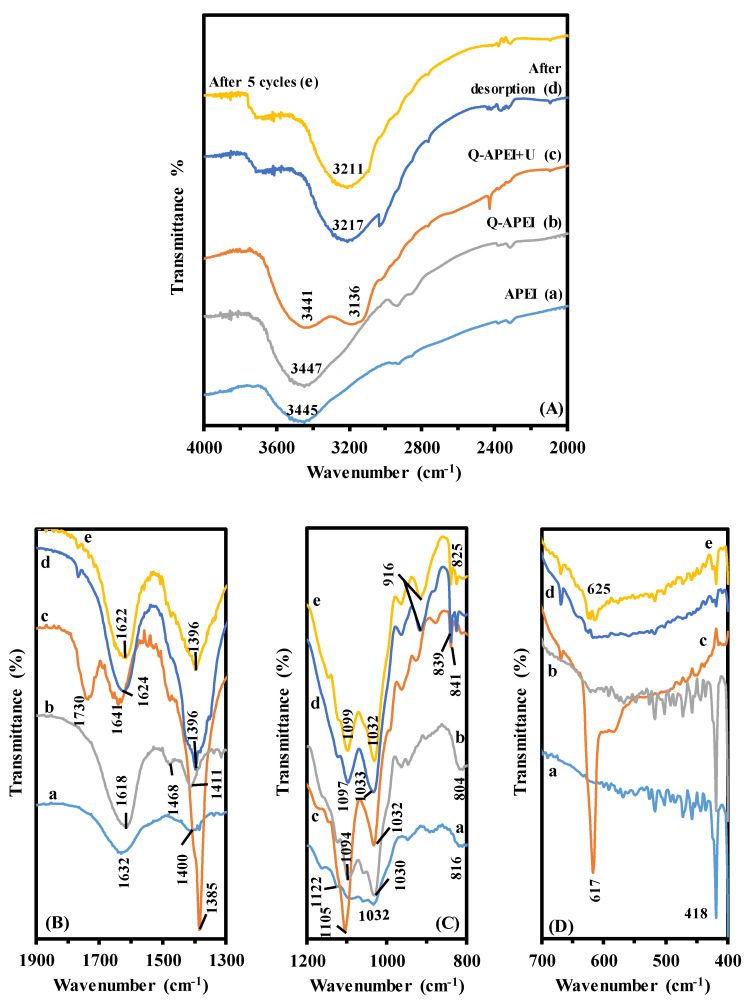
FTIR spectra (selected wavenumber ranges).

The composition of the sorbent based on algal biomass, alginate and PEI offers a large variety of functional groups: -NH, -OH, and –COOH. The presence of these groups can be identified on the FTIR spectra of APEI and Q-APEI (Figure 1). The assignment of main peaks are reported on Table S1 (see Supplementary Information). The sorption of uranyl is followed by modifications of the characteristics bands: these changes help in understanding the functional groups involved in metal binding. The figure also shows the FTIR spectra for the sorbent after uranyl desorption (to check the restauration of functional groups) and after five cycles of sorption and desorption (to evaluate the stability of the sorbent). 

The first panel on Figure 1 shows the region where the stretching vibrations of –OH and –NH groups are identified (overlapping). The broad band around 3445 cm^−1^ is not significantly affected after quaternization (around 3447 cm^−1^). Peaks appearing around 2854 and 2930 cm^−1^ are assigned to C-H stretching in alkane and alkene compounds. Two small peaks appear at 2328 cm^−1^ and 2374 cm^−1^; they may be attributed to O=C=O stretching in carbon dioxide. Wang et al. [42] reported the sorption of CO_2_ on similar PEI-algal based materials. After uranyl sorption, substantial changes are observed: a new broad band appears at 3116 cm^−1^. This is probably associated with the interaction of uranyl ions with hydroxyl groups and especially amine groups. The C-H stretching vibration poorly appears (due to overlapping effects of the new broad band). The band around 2350 cm^−1^ also disappears. The small peaks at 2328 and 2374 cm^−1^ re-appear after desorption (first cycle and fifth cycle). After regeneration of the resin, the two bands at 3441 and 3136 cm^−1^ are displaced and merged in a single broad band in the range 3217–3211 cm^−1^. This is a confirmation of the strong implication of –NH and –OH groups in the interactions of uranyl ions with the sorbent. In addition, the analysis of regenerated sorbent shows the appearance of a new poorly resolved band at around 3700 cm^−1^, this band can be assigned to alcohol functions. Therefore, the regeneration of the sorbent alters the chemical structure of the sorbent. It will be important checking how this modification can interfere with sorption performance. 

A broad band appears at 1632 cm^−1^ on APEI (1618 cm^−1^ on Q-APEI), probably as the convolution of two signals corresponding to N=C stretching (associated with the cross-linking of aldehydes on GA (glutaraldehyde) and amine groups on PEI) and C=O stretching. After uranyl sorption, a new strong peak appears around 1730 cm^−1^ (with a decrease in the width of the band at 1632–1618 cm^−1^). This peak is usually assigned to C=O stretching in carboxylic acid and ketone groups: the binding of uranyl ions affects amine groups and modifies the spectrum to shows carboxylate-like band. The peak at 1385 cm^−1^ is also strongly marked after uranyl binding, this is a representative peak of O-H bending vibration. After metal desorption and sorbent recycling, the peak at 1730 cm^−1^ disappears and the intensity of the peak at 1385 cm^−1^ strongly decreases: the sorbent is fully regenerated at the level of amine groups. Metal sorption is also marked by the formation of a strong peak at 1105 cm^−1^, which can be attributed to C-O stretching in secondary alcohol. The intensity of this peak decreases after metal desorption and sorbent recycling but it does not disappear. The peak at 841 cm^−1^, which appears after uranyl binding, is directly associated with the O=U=O vibration [43,44]. A new strong band is also observed at 617 cm^−1^ when uranium is sorbed and usually assigned to sulfate groups. This is a first indication that uranyl is probably bound to Q-APEI under the form of uranyl sulfate complex, or at least that sulfate anions are bound on quaternary ammonium groups of Q-APEI. After metal desorption, the strong peak disappears: sulfate being exchanged with chloride ions and being released through the desorption of uranyl sulfate ions.

#### 2.1.2. XPS Analysis

**Figure 2:**
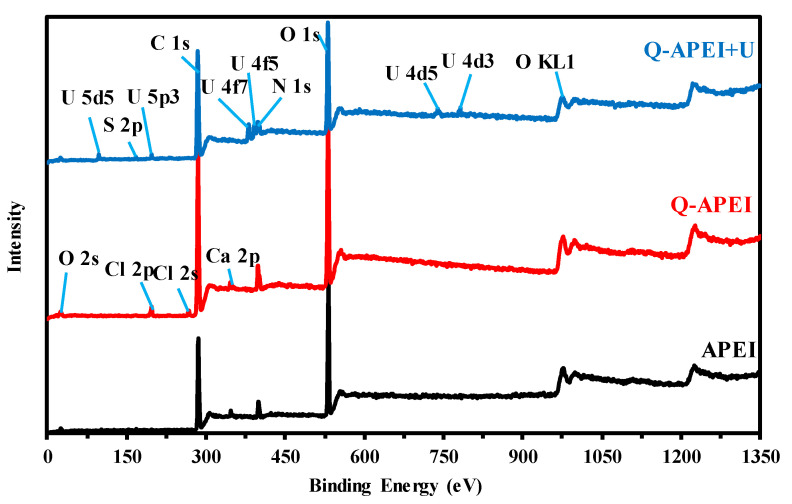
XPS survey of raw material (APEI), after modification (Q-APEI), and after uranium sorption (Q-APEI-U).

XPS analysis was also used for characterizing metal binding. XPS survey spectra for APEI and Q-APEI (before and after U(VI) sorption) are reported on Figure 2. The main elements are identified associated with APEI: organic fraction containing C, O, and N elements. After quaternization, the intensity of N *1*s is more marked, and chlorine element appears (Cl^−^ counter anions on quaternary ammonium groups, Cl *2*p and Cl *2*s). After uranyl sorption, chlorine element disappears being replaced with S element (S *2*p, for example) and a series of peaks assigned to U elements are identified: U *5*d_5_, U *5*p_3_, U *4*f_7_, U *4*f_5_, and U *4*d_5_. The appearance of S *2*p signal confirms the binding of sulfate anions directly on quaternary ammonium groups on the sorbent, through anion-exchange with Cl^−^ anions. The disappearance of calcium (i.e., Ca *2*p) probably means that a fraction of uranyl (essentially free cationic uranyl; i.e., UO_2_^2+^) may be bound on carboxylate groups by cation-exchange with calcium.

High-resolution spectra (HRES) (and the assignments of deconvoluted peaks) for selected signals are also shown in Tables S2 and S3. These data were previously discussed [41]. Tables S4 and S5 discuss the HRES of XPS spectra of relevant signals after uranyl binding.

The C *1*s signals splits into four deconvoluted peaks after U(VI) binding, these peaks are different than in the XPS spectra of APEI and Q-APEI with overlapping of C(=O, -O-C) and O-C(=O, C-O) peaks at a binding energy (BE) (eV) close to 286.97 eV. This is followed by a significant increase in intensity or atomic fraction (AF) from 0.72% and 7.75% in APEI and Q-APEI, respectively, to 15.57% after uranium binding. Other peaks corresponding to C (C, N, H), C(-NH or NH_2_), and C(-O, =N) shift to lower binding energies (compared with APEI and Q-APEI) at BEs: 283.9 eV, 284.58 eV, and 285.85 eV, respectively [45,46,47].

N *1*s signal appearing as the convolution of three peaks corresponding to N-C, N-H and N=C is shifted to 398.43 eV (compared with 399.2 eV for APEI, and 398.44 eV for Q-APEI). The signal representative of N_tert._ [48] shifts to higher binding energy compared with Q-APEI after uranyl sorption. This means that a charge transfer occurs between uranyl ions and N-containing ligands [49]. N_tert_ is shifted from 400.74 eV in APEI, to 399.7 eV in Q-APEI and to 398.43 eV for N-U interaction [50,51]. The BE of N^+^ in quaternary ammonium groups is weakly shifted after metal binding (from 401.55 to 401.65 eV).

Strong differences are observed in the profiles of O *1*s when comparing uranyl-bonded sorbent with the spectra of APEI and Q-APEI. The signal represents the overlapping of C=O, C-O, and O-H, which appears at 531.09 eV [52,53,54,55,56] (instead of two peaks at around 530.5 and 532 eV in APEI and Q-APEI). The disappearance of O-Ca peak confirms the partial ion-exchange of Ca(II) with uranyl cation (on carboxylate groups); the peak at 529.92 eV is assigned to U=O [49,57,58]. 

**Scheme 1:**
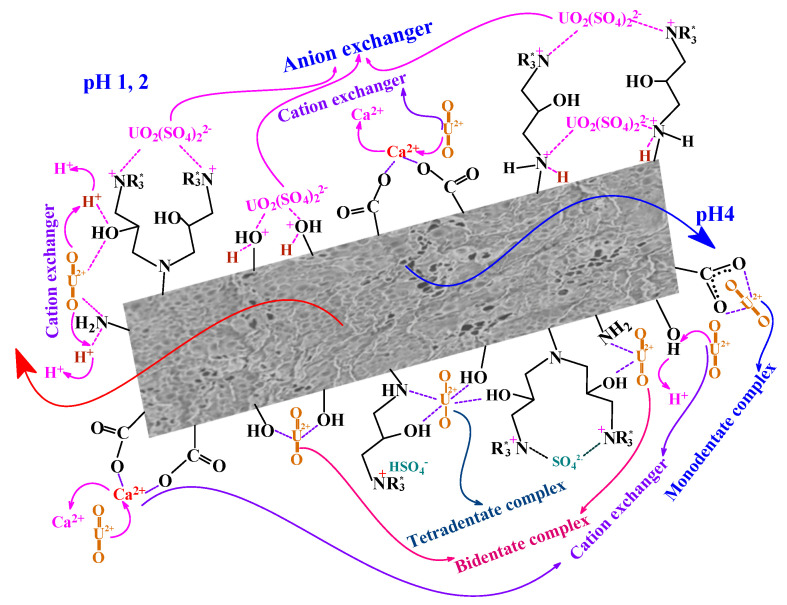
Tentative mechanism for U(VI) sorption on Q-APEI.

As reported above, the appearance of S *2*p signal on metal-loaded sorbent at 167.25 eV (which is assigned to sulfate groups [59,60,61,62]) means that sulfate (as well as HSO_4_^−^) probably binds onto the quaternary ammonium groups at the surface of the sorbent at pH 4 (see Scheme 1, part corresponding to pH 4). 

The signals associated with Ca *2*p (i.e., Ca *2*p_3/2_ and Ca *2*p_1/2_) are significantly affected by metal sorption both in terms of BEs, full width at half maximum (FWHM) values and atomic fractions (Tables S2–S5). This confirms the ion-exchange of calcium (involved in ionotropic gelation of carboxylic groups on alginate) with cationic uranyl species for binding on carboxylate groups. 

U *4*f is deconvoluted into four peaks: U *4*f_5/2_, which splits into two peaks at 389.71 eV and 391.34 eV, while peak at 380.23 eV is assigned to U *4*f_7/2_, this attributed to free uranyl adsorption and covalent bond of N-U(VI) sorption [63]. The other peak at 384.31 eV corresponds to the satellite peak and confirms that U is present under its +6 oxidation state. The deconvolution of U *4*d signal shows five splitting peaks at 738.35 eV, 739.85 eV, 737.4 eV, 736.45 eV, and 742.9 eV. Other peaks, at 1272.31 eV and 1048.93 eV, correspond to U *4*p1 and U *4*p3, respectively. 

These results suggest that sorption occurs through different mechanisms:

(a) anionic exchange between Cl^−^ ions on quaternary ammonium groups and anionic uranyl species [64] (at pH 1–2),
(b) ion-exchange with Ca^2+^ bound to carboxylate groups, protons on the hydroxyl and amine groups and cationic UO_2_^2+^ (especially at pH < pH_PZC_ corresponding to particle deprotonation), and/or
(c) complexation of metal species with free nitrogen or oxygen donors from PEI, hydroxyl moieties created from opened epoxy groups, and polysaccharide moieties, respectively.

#### 2.1.3. Determination of pH_PZC_

Figure S1 (see Supplementary Information) shows the application of the pH-drift method for the evaluation of pH_PZC_ of APEI and Q-APEI. As expected, the pH_PZC_ significantly increases with quaternization from 4.82–5.05 (APEI) to 6.72–6.76. The quaternization allows maintaining a positively-charged surface of the sorbent on a wider pH range, at least the pH range where uranyl sorption will be processed. At high pH values (except when complexed with carbonate, for example) uranyl species may precipitate (above pH 5.5 for concentrations around 100 mg U L^−1^; 0.42 mmol U L^−1^). The comparison of recorded pH variations shows that the pH change is larger (up to 0.7 pH unit) for Q-APEI below its pH_PZC_ compared with APEI (down to 0.3 pH unit). Above pH_PZC_, the pH variation is more marked for APEI (up to 2.6 pH units at pH 6). The proton exchange properties are logically strongly affected by the quaternization of amine groups. This also means that the protonation of the sorbent will make easier the sorption of anionic metal species if present in the whole pH range.

The sorption is essentially constituted of alginate (alginate extracted from algal biomass, completed by the addition of supplementary alginate), polyethylenimine, and glycidyltrimethylammonium chloride. The main reactive groups present at the surface of the sorbent are:

(a) carboxylic groups (mannuronic and guluronic acid with pK_a_ values of 3.38 and 3.65, respectively, [65]),
(b) amine groups (primary, secondary, and tertiary (1/2/1) with pK_a_ values of 4.5, 6.7, and 11.6, respectively, [66]), and
(c) quaternary ammonium groups (with pK_a_ in the range 11–12).
(d) hydroxyl groups

Therefore, the global charge can be disconnected to the individual acid–base contributions of individual reactive groups, and their potential interactions with metal species. The overall charge plays on the global properties of attraction/repulsion of sorbent surface for charged metal ions.

### 2.2. Uranium Sorption

#### 2.2.1. pH Effect

**Figure 3:**
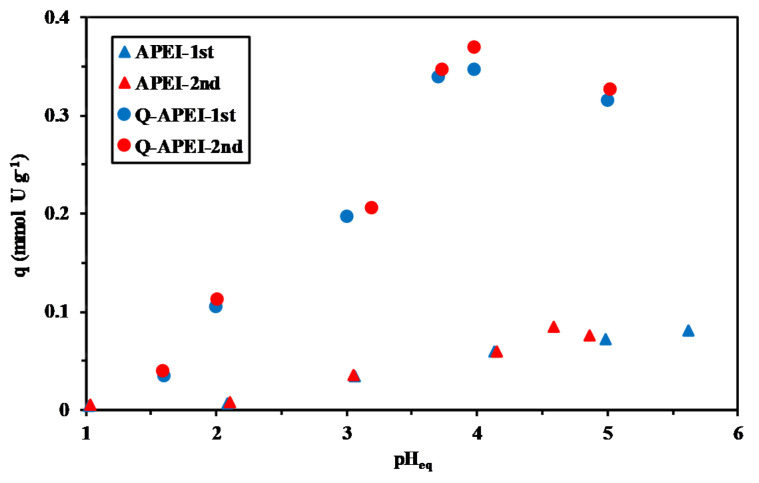
Effect of pH on U(VI) sorption using APEI and Q-APEI sorbents (Sorbent dosage (SD): 0.333 g L^−1^; contact time: 48 h; agitation speed: 170 rpm; T: 22 ± 2 °C).

The quaternization strongly increases the efficiency of the sorbent for binding uranyl ions in the full range of pH (Figure 3). The superimposition of the curves shows the good reproducibility of sorption performances. Under identical experimental conditions, the sorption capacity never exceeds 0.085 mmol U g^−1^ for APEI, while for Q-APEI, the sorption capacity reaches up to 0.35 mmol U g^−1^ at the optimum pH_eq_ (i.e., 4). Despite the presence of amine groups, hydroxyls and carboxylic groups on APEI, both the crosslinking of amine groups with glutaraldehyde, and the possible interactions between protonated amine groups and carboxylic groups contribute to reduce the reactivity of functional groups. The quaternization increases the density of amine groups (from 4.15 to 6.12 mmol N g^−1^) [41]. Branched PEI is constituted of primary/secondary/tertiary amine groups (according to the distribution: 1/2/1, with pK_a_ values: 4.5, 6.7, and 11.6, respectively [66]). Below pH 4, all of the amine groups are protonated but the steric hindrance (associated with specific interactions of PEI with glutaraldehyde and/or carboxylic groups of alginate) may limit their reactivity. The positively-charged surface of the sorbent on the whole pH range enhances the sorption of anionic species. At pH 1, the competition of counter anions in the solution makes negligible uranyl binding. With increasing the pH, the sorption capacity linearly increases: the competition of dissociated anions progressively decreases and sorption of uranyl is enhanced. 

Figure S2 shows the log_10_ plots of distribution ratio (D: q_eq_/C_eq_, L g^−1^) vs. equilibrium pH, for both APEI and Q-APEI. The curve for Q-APEI is about one order of magnitude higher than for APEI, this is a confirmation of the higher affinity of quaternized sorbent for uranyl. The two curves show two linear regions: the slope changes around pH 3.5–4. In the acidic region, the slope is close to 0.42 for the two sorbents. This means that the binding of one uranyl will probably involve the exchange of two protons. Above pH 3.5–4, the linear correlations are less marked and the slopes tend to decrease. When pH exceeds 4, the sorption capacity tends to slightly decrease.

Figure S3a shows the speciation diagram for uranyl ions (under the experimental conditions selected for the study of pH effect). Precipitation may occur at pH higher than 5. Further experiments have been performed at pH 4 (optimum pH, with no precipitation of U(VI)). At low pH (i.e., pH 1), uranyl is essentially present as a neutral species (i.e., UO_2_SO_4_, ≈ 65%), while cationic free UO_2_^2+^ represents about 21% and anionic species (i.e., UO_2_(SO_4_)_2_^2−^) counts for less than 14%. As the pH increases (above pH 2 and up to pH 4), anionic uranyl species disappears, and the fraction of neutral uranyl sulfate strongly decreases while progressively uranium dioxide begins to predominate (up to 73%). Above pH 4 and up to pH 5 (where U(VI) begins to precipitate) free uranyl fraction strongly decreases while hydrolyzed and polynuclear hydrolyzed species appear (mainly as (UO_2_)_2_(OH)_2_^2+^, (UO_2_)_3_(OH)_5_^+^, UO_2_(OH)^+^, or (UO_2_)_4_(OH)_7_^+^). The increase in sorption properties with pH up to 4, the stoichiometric exchange (i.e., slope of log_10_ D *vs.* pH_eq_), the predominance of free uranyl species (UO_2_^2+^) in this pH range, tend to demonstrate that uranyl sorption occurs through ion exchange of UO_2_^2+^ with protons on amines and hydroxyls groups. The presence of sulfate on the sorbent (identified by XPS analysis) is probably associated to the direct binding of sulfate anions onto quaternary ammonium groups or through interaction with uranyl bound to the sorbent.

The variation of pH with uranyl sorption (as appearing on Figure S4) is negligible for APEI sorbent up to pH 5. Above pH 5, uranyl precipitation logically causes pH decrease. For Q-APEI sorbent, pH variation is negligible between pH 2 and 3 and above pH 3, the sorption of uranyl tends to decrease the pH due to proton release (proton exchange with uranyl) and the formation of hydrolyzed species (which may be bound on the resin).

Table S6 compares the semi-quantitative EDX analyses of Q-APEI loaded with uranyl at different pH values. In strong acid solutions (i.e., pH 1 controlled with sulfuric acid) the sorbent is strongly protonated with relatively high atomic fractions for Cl (i.e., 2.56%) and S ((i.e., 8.6%, as sulfate) while U content does not exceed 0.3%. With the pH augmentation, the U content progressively increases up to 2.79%–2.9% at pH 4 and pH 5, respectively. At pH 6, U content decreases to 0.92%. The level of Cl slightly varies between 0.5% and 0.84% while S content remains stable (around 3.0%–2.7%) between pH 2 and 5 and decreases to 1.9% at pH 6. Sulfate content varies due to direct binding on protonated groups in acidic solutions, while at intermediary pH the presence of sulfate is probably associated with the binding of uranyl sulfate or the binding of sulfate on uranyl bonded on the sorbent.

#### 2.2.2. Sorption Mechanism

From the data of FTIR, XPS, slope analysis of log_10_ D *vs.* pH_eq_, and sorption profiles, the suggested mechanism can be discussed at two different pH values: acidic pH (at pH 1–2) and mild acidic medium (at pH 4). At pH 1–2, the speciation of uranyl shows the predominance of anionic uranyl sulfate species ((UO_2_(SO_4_)_2_^2−^), which could be sorbed onto the sorbent through anion-exchange with chloride ions bond on quaternized sites, on protonated amines and hydroxyls protonated groups. This electrostatic attraction mechanism can explain the relatively higher sorption capacities observed for Q-APEI compared with APEI. On the other hand, the cationic free uranyl species binds with the sorbent by cation exchange with protons (from amines, and hydroxyls) and Ca^2+^ ions from carboxylate groups (see Scheme 1). At pH 4, the sorption of U(VI) onto Q-APEI might be confirmed by either complexation reaction through electron donating acceptor (mono-, bi-, and tetradentate complexes) and cationic exchanger mechanism depending on the groups on the sorbent and the pH. At experimental pH values, the main uranyl species found in the solution is UO_2_^2+^ (and other species with lower percentage, such as UO_2_OH^+^, (UO_2_)_2_(OH)_2_^2+^). The negatively charged uranyl species (UO_2_(SO_4_)_2_^2−^) becomes progressively negligible at higher acidic pH values. 

From the FTIR analysis of Q-APEI, before and after loading with uranyl ions, it is evident that the main sorption mechanism performed with OH, NH, and COO^−^ by complexation reaction and with QA groups by anion exchange reaction. FTIR analysis of loaded sorbent shows that the broad band (overlapping of hydroxyl and amine stretching vibrations) shifts from 3445 to 3136 cm^−1^. A new peak also appears at 3441 cm^−1^. These observations confirm that OH and NH groups contribute to U(VI) sorption. The peak assigned to carboxylic acid is shifted from 1385 to 1411 cm^−1^, meaning that the environment of carboxylic groups is also affected by uranyl binding. The changes in the environment of C=N (shifted from 1618 to 1641 cm^−1^) and the appearance of another peak, assigned to C=O of carboxylic acid salt, at 1730 cm^−1^, emphasize the contribution of these groups in uranyl binding. This is also confirmed by the shifts of C-O and C-N peaks from 1094 and 1030 cm^−1^ to 1105 and 1032 cm^−1^ after metal uptake. The new peak appearing at 617 cm^−1^ can be attributed to sulfate groups: this may proceed through the binding of uranyl sulfate complex and the direct binding of sulfate or hydrogen sulfate ions onto quaternized groups.

From the XPS survey of loaded sorbent, the disappearance of Ca and Cl confirm the ion-exchange mechanisms, as reported above. The splitting of U *4*f to U *4*f_5/2_ (two splitting peaks) and U *4*f_7/2_, at lower values than usual, confirms that two types of binding occurred on the sorbent (probably through the formation of U-N and U-O bonds). This may be correlated to the shifts of the N_tert_ and N^+^ peaks associated with either complexation and anion exchange mechanisms, respectively.

#### 2.2.3. Uptake Kinetics

**Figure 4:**
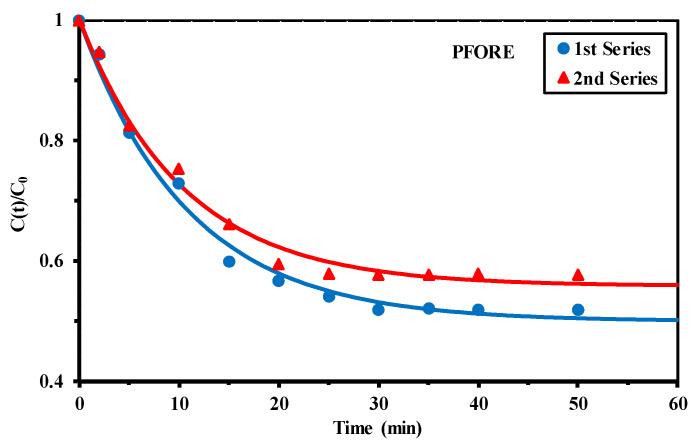
U(VI) uptake kinetics using Q-APEI sorbent—Modeling with the pseudo-first order rate equation (PFORE) (SD: 0.3 g L^−1^; pH_0_: 4; pH_eq_: 3.79–3.71; C_0_: 0.214 mmol U L^−1^ and 0.249 mmol U L^−1^ for the 1st and 2nd series, respectively; agitation speed: 170 rpm; T: 22 ± 2 °C).

Figure 4 shows that the equilibrium is reached under selected experimental conditions within 30–40 min. The mesoporous structure of the sorbent [41] may explain the readily transfer of metal ions through the sorbent. Sorption may be controlled by the resistance to film diffusion, the resistance to intraparticle diffusion but also by the proper reaction rate. Table S7 reports the equation of these models. 

The fast kinetics suggests that the resistance to intraparticle diffusion does not represent the main controlling step. In Figure 4, the solid lines show the fits of experimental profiles with the pseudo-first order rate equation (PFORE); the modeling of kinetics with the PSORE (pseudo-second order rate equation) and RIDE (resistance to intraparticle diffusion equation—Crank equation) are represented in Figure S6. The PSORE fails to fit the equilibrium: overestimation of the equilibrium time with the sorption continuing above 50 min. This is confirmed by the large difference in the experimental and calculated (overestimated) values of the sorption capacity at equilibrium (Table 1). The RIDE (i.e., Crank equation) generally respects the shape of kinetic profiles, but the determination coefficients are much smaller than those obtained with the PFORE. However, this model can be used for evaluating the diffusion coefficient (D_eff_, m_2_ min^−1^) for uranyl through Q-APEI: D_eff_ varies between 2.6 × 10^−8^ and 3.3 × 10^−8^ m^2^ min^−1^. These values are comparable to the free diffusivity of uranyl in water (i.e., 2.56 × 10^−8^ m^2^ min^−1^, [67]). This confirms the weak impact of resistance to intraparticle diffusion on the control of kinetic profiles and this is fully consistent with the average size of pores (i.e., 183–230 Å, [60]), which is much larger than the size of hydrated uranyl (UO_2_(H_2_O)_5_^2+^, 1.08 Å). The PFORE slightly overestimates the sorption capacity at equilibrium: 0.358–0.365 vs. 0.343–0.349 mmol U g^−1^.

The apparent rate coefficient is in the range: 9.19–9.61 × 10^−2^ m^2^ min^−1^. The preference of the fit of experimental kinetic profile with the PFORE (compared with pseudo-second order rate equation, PSORE) is consistent with the suspected nature of the interaction mode (ion-exchange compared chelation mechanism).

#### 2.2.4. Sorption Isotherms

**Figure 5:**
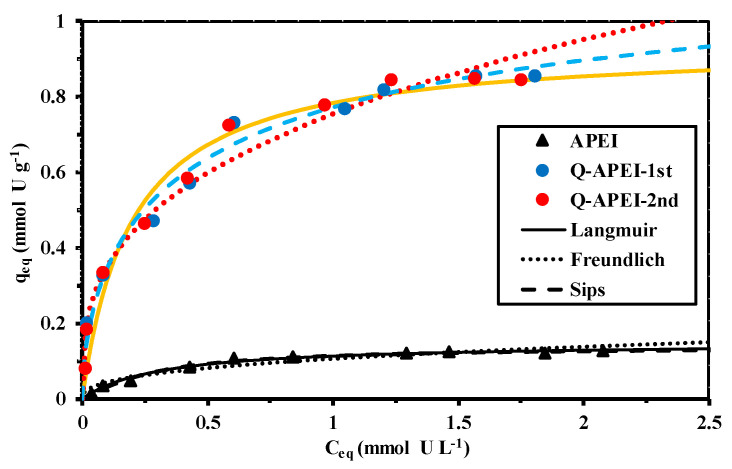
U(VI) sorption isotherms at pH 4 using APEI and Q-APEI—Modeling with the equations of Langmuir, Freundlich, and Sips (SD: 0.375 g L^−1^; pH_0_: 4; pH_eq_: 3.87–3.69 for Q-APEI and 4.19–3.97 for APEI; C_0_: 0.04–2.13 mmol U L^−1^; agitation speed: 170 rpm; contact time: 48 h; T: 22 ± 2 °C).

Figure 5 compares U(VI) sorption isotherms at pH 4 for APEI and Q-APEI sorbents. Sorption capacity is plotted as a function of residual metal concentration in the solution. The experimental profiles are fitted with the Langmuir, Freundlich, and Sips equations. Table S8 summarizes the equations used for modeling sorption isotherms. Table 2 summarizes the parameters of the models used for fitting sorption isotherms. The shape of the sorption isotherms are characterized by a steep initial slope followed a plateau. This asymptotic trend is consistent with the Langmuir and Sips equations, contrary to the Freundlich equation that supposes an exponential trend. This is confirmed by the poor fit of experimental profile at high residual metal concentration (overestimation above 1.3 mmol U L^−1^), and in the intermediary range of concentration (underestimation between 0.42 and 1 mmol U L^−1^). In the case of APEI, the fitted curves approaches much better the experimental profile. The Sips equation incorporates a third-adjusting parameter. It is thus expected that the three-parameter equation best fits experimental data. Actually, this is not really the case here: the improvement in the quality of the fit with the Sips is not significant.

The sorption of uranyl is strongly improved by quaternization: maximum sorption capacity increases from 0.13 to 0.85 mmol U g^−1^. This six-fold increase clearly demonstrates the highly efficient functionalization of the support, which is not simply correlated to the increase of amine groups (from 4.15 to 6.12 mmol N g^−1^; i.e., about + 50%). At saturation of the sorbent, the molar ratio between U and N is close to 0.139. This molar ratio is not consistent with the expected stoichiometric ratio (two protons from reactive groups per bound uranyl). This means that all the amine groups are not involved in metal sorption. The increase in nitrogen after quaternization is close to 2 mmol N g^−1^. Taking into account the stoichiometric molar ratio for the interaction of uranyl with the sorbent (i.e., two protonated amine groups per bound uranyl) this means that, at saturation, the ratio 2:1 is respected for the binding of one uranyl to two quaternary ammonium groups immobilized per gram of sorbent. It is noteworthy that the affinity coefficient (i.e., b_L_) is higher for Q-APEI compared with APEI: the quaternization improves the efficiency of the sorbent not only in terms of maximum sorption capacity (asymptote of the sorption isotherms) but also in terms of affinity (initial slope of the curve).

Table 3 compares the sorption performance for U(VI) using different sorbents. The sorbent Q-APEI shows sorption properties comparable to those of the most efficient sorbents taking into account both the kinetic and the equilibrium criteria. Some specific new resins (functionalized with picolylamine groups, for example, [68]) and amidoximated sorbents [31,69,70] show higher maximum sorption capacities. Except these highly-efficient sorbents, Q-APEI shows promising properties. The ability of the metal-loaded material to be eluted and the sorbent to be recycled should be demonstrated for justifying these perspectives. It is noteworthy that FTIR analysis after five cycles of sorption and desorption showed some punctual differences but the general structure of the sorbent did not appear to be drastically changed.

#### 2.2.5. Uranium Desorption and Sorbent Recycling

**Figure 6:**
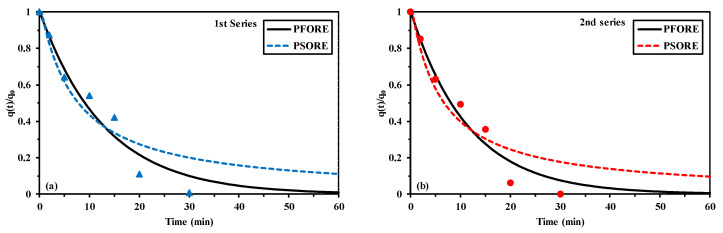
Desorption kinetics for U(VI) loaded on Q-APEI—Modeling with the PFORE and the PSORE for the 1st series (**a**) and the 2nd Series (**b**) (samples collected from uptake kinetics; eluent: 0.5 M NaCl/0.5 M HCl; SD: 0.85 g L^−1^; agitation speed: 170 rpm; T: 22 ± 2 °C).

In order to elute uranium from metal-loaded sorbent, an acidic solution of sodium chloride was selected. The batch desorption of uranyl ions was tested for evaluating desorption kinetics (Figure 6 and Table 4) and recycling performances (Table 5). Metal desorption is a fast process: 30 min of contact are sufficient for achieving the complete desorption of uranyl, under selected experimental conditions. These kinetic profiles are slightly more favorable than the uptake kinetics. The kinetic profiles are finely fitted by the PFORE (Table 4), the PSORE–simulated curve does not reach the complete desorption of uranyl. The apparent rate coefficient for desorption (according PFORE model) is of the same order of magnitude than the corresponding apparent rate coefficient for sorption: around 0.08 min^−1^ vs. around 0.09 min^−1^, while the SD was substantially increased 0.85 g L^−1^ compared with uptake kinetics (i.e., 0.3 g L^−1^). This confirms that the main mechanisms of desorption (as well as sorption) is associated to an ion-exchange process.

Table 5 confirms the remarkable stability of the sorbent in terms of sorption and desorption efficiencies. A very limited decrease is observed for uranium uptake (less than 1%) and metal desorption (less than 2%). This confirms the efficient regeneration of the sorbent after one and five cycles as shown by FTIR characterization (Figure 1).

### 2.3. Application to Uranium-Bearing Ores: Treatment of Acid Leachates

The resin was tested for a very complex effluents obtained from acidic leaching of carbonaceous shale collected at the Allouga mining site. 

#### 2.3.1. Acid Leaching of Ore and Pre-Treatment

Table S9 reports the concentrations of the major elements in the acidic leachate (PLS). Copper and iron represent the most abundant metals: 34 g Cu L^−1^ and 6.5 g Fe L^−1^, respectively. Other valuable metals are also present at exploitable levels: 600 mg U L^−1^ and 220 mg REE L^−1^. The large excess of base metals limits the possibility to recover the valuable traces of U and REEs. It is thus necessary pre-treating the PLS. Copper was separated by cementation and the analysis of the filtrate after cementation shows a low residual Cu concentration (close to 94 mg Cu L^−1^): copper recovery reaches up to 99.6%. Uranium loss reaches 25%, while REEs remain almost unchanged (loss below 2.7%). Obviously, the cementation using iron powder leads to an increase of iron concentration (up to 7.7 g Fe L^−1^). The semi-quantitative EDX analysis of the copper-cake shows the predominance of three elements: Cu (39.24%, atomic fraction), S (38.38%), and Fe (17.45%) (Figure S6). The contamination of the copper-cake consists of Si (2.97%), P (1.86%), and traces of U (0.1%).

Due to the large excess of iron in the treated PLS, a second pre-treatment was applied that consisted of the removal of Fe by precipitation. The pH was raised to 5 by NaOH. The semi-quantitative EDX analysis of the iron precipitate shows that the precipitation is selective as Fe(OH)_3_; the presence of sulfur is due to highly sulfate medium from sulfuric acid pug leaching (Figure S7).

The filtrate was then treated with oxalic acid for selective precipitation of REEs oxalate. Table S9 reports the analysis of the solution after iron precipitation and REEs selective precipitation. These complementary treatments reduce the residual concentration of copper to 3 mg Cu L^−1^, while the concentration of REEs decreases to 5.3 mg L^−1^ (97.5% of REEs are removed after oxalate treatment). The yield of iron precipitation reaches 75%: residual concentration remains close to 1925 mg Fe L^−1^. After this series of treatments, uranium concentration reaches 350 mg U L^−1^; the loss reaches 22.3% after iron and REEs precipitations. After the Cu cementation and the successive precipitation steps, the loss of uranium reaches 42%.

#### 2.3.2. Metal Sorption from Leachates and Uranium Recovery

Figure S8 shows the kinetic profiles for the sorption of U(VI) at pH_0_ 2 and 4 (pH_eq_: 2.98 and 3.81, respectively). The kinetics are much slower with these complex solutions than with synthetic solutions: while less than 1 h was sufficient for reaching the equilibrium, with pre-treated PLS, a contact time of 24 h is necessary. As expected, the sorption efficiency is substantially enhanced at pH 4 (58% instead of 23% at pH 2). The sorption capacity reaches 0.21 mmol U g^−1^ (compared with 0. 84 mmol U g^−1^).

Figure S9 compares the distribution ratios (D, L g^−1^) for Cu, Fe, and U, for the two solutions. The values of D are very low for Cu and Fe (systematically below 0.01 L g^−1^), while for U the D value ranges between 0.07 and 0.35 L g^−1^ (from pH 2 to pH 4). The relevant sorption capacities show negligible sorption of copper (very weak initial concentration) and comparable sorption capacities for iron (in very large excess) and uranium. Despite the 34-fold excess of iron compared with uranium in the pre-treated PLS, the sorption of uranium reaches very high accumulation. Though the sorption is not selective, the sorption on Q-APEI allows enriching preferentially the resin with uranium. This is confirmed by the determination of the selectivity coefficients (SC_U/metal_: D_U_/D_metal_) (Figure S10). Despite the large excess of iron, the SC_U/Fe_ is independent of the pH and close to 40. Q-APEI is very efficient for U sorption in complex solutions. The sorbent cannot separate U from iron but the strong efficiency of the sorbent for uranium explains the high levels of accumulation of the metal on the sorbent.

The metals bound onto the sorbent (loaded at pH 2 and 4) are eluted using HCl solution. The eluates are treated by precipitation using NaOH solution (pH controlled to 9). The yellow cake (probably sodium diuranate, Na_2_U_2_O_7_ is collected by filtration, after washing several times with water for removing NaCl, after drying, it was semi-quantitatively analyzed by EDX. Figure S11 shows that the precipitate obtained from sorbent loaded at pH 2 contains a wide range of metals and elements (including iron, phosphorus, sodium, aluminum, calcium, silica, and chloride) with relatively low U atomic fraction (i.e., 8.78%). On the opposite hand, the sorption at pH 4 being more selective for U, logically the relevant eluate contains a much higher fraction of U (AF: 55.34% and weight fraction: 51.6%) and the impurities represent less than 8.3% (AF) and 3.3% (weight fraction).

## 3. Conclusions

In order to increase the sorption of uranyl ion, a new generation of composites made by the interaction of alginate (from algal biomass) and polyethylenimine (ionotropically gelled with CaCl_2_ and cross-linked with glutaraldehyde, respectively) is successfully quaternized by reaction with glycidyltrimethylammonium chloride. The quaternization shifts the pH_PZC_ of the sorbent by 1.7 pH units: the sorbent is fully protonated on the whole range of pH used for U(VI) sorption (below precipitation). The increased density of positively-charged sites enhances uranyl sorption that can reach 0.85 mmol U g^−1^ at the optimum pH (i.e., pH 4). Sorption occurs by a combination of mechanisms, whose relative contributions depend on the pH and metal speciation, including: chelation of positively species on amine groups, binding of anionic species on protonated amine and quaternary ammonium reactive groups, binding on hydroxyl and carboxylate groups. The binding of sulfate anions may introduce some competition effects (the binding of sulfate anions is clearly identified by different techniques). These interactions are identified through FTIR and XPS analyses. Sorption is a relatively fast phenomenon: 40 min are sufficient for reaching equilibrium. The kinetic profiles are best fitted by the pseudo-first order rate equation (PFORE) while sorption isotherms are described by the Langmuir equation. Uranyl desorption is successfully operated using acidic NaCl solutions (0.5 M NaCl/0.5 M HCl): desorption kinetics (also fitted by the PFORE) is even faster than the sorption kinetics. The conditions for metal desorption are highly efficient (yield close to 100%) and the sorbent can be re-used for a minimum of five cycles with negligible loss in efficiencies for both sorption and desorption; this is confirmed by the stability of FTIR spectra after five operating cycles. Acidic leachates of polymetallic carbonaceous shale are pre-treated for separating copper (by cementation) and rare earth elements (by oxalic acid precipitation). Though a precipitation step (adjusting the pH to 5) allows removal of an important fraction of iron, the residual concentration of Fe remains about 34-fold higher than U(VI) concentration. Despite this large excess, the sorbent can readily adsorb U(VI) and a yellow cake (sodium diuranate) is obtained after precipitation of the eluate. The grade of impurities in the yellow cake is much lower for sorbent processed at pH 4. 

Conventional resins are usually applied in dynamic systems using fixed-bed reactors. Therefore, complementary work would be necessary to evaluate the behavior of Q-APEI beads in columns and more specifically to compare the sorption capacities obtained at saturation of the bed with the sorption capacities reported in batch systems. The packing of Q-APEI beads in high column reactor is expected to produce important mechanical constraints. Therefore, it would be also useful evaluating the physical stability of the material under compression, especially along successive cycles of desorption and desorption.

## 4. Materials and Methods

### 4.1. Materials

Algal biomass (*Laminaria digitata*) was provided by Setalg (Pleubian, France). The biomass was grinded and the fraction below 250 µm was collected for processing the synthesis of the sorbent. Alginate (Manugel GMB) was supplied by FMC (Landerneau, France; now JRS Rettenmaier). This alginate has the following specifications: water content 16.3% (by TGA), MW 446,000 g mol^−1^ (viscosimetric measurements and Mark Houwink Sakurada equation) and G/M ratio 0.19/0.81 (by ^1^H NMR analysis). Polyethylenimine (branched PEI, 50% *w/w*) was purchased from Sigma-Aldrich (Taufkirchen, Germany). Ethylene glycol diglycidyl ether, glycidyltrimethylammonium chloride (≥95%) and dimethylformamide (DMF) were purchased from Shanghai Makclin Biochemical Co., Ltd. (Shanghai, China). Na_2_CO_3_ and CaCl_2_ were obtained from Chem-Lab NV (Zedelgem, Belgium). Uranyl sulfate (single-metal experiments) was supplied by Polysciences Europe GmbH (Hirschberg an der Bergstraße, Germany).

### 4.2. Sorbent Synthesis

The first step in the process consists of the particle extraction of alginate contained in algal biomass by a thermal alkaline extraction: algal biomass (9.375 g) was mixed for 24 h at T: 50 °C in 375 mL of sodium carbonate (1% *w/w*). Preliminary experiments showed that depending on the mode of functionalization, the addition of a small amount of alginate contributes to reinforce the stability of the sorbent. After alkaline thermal extraction, the suspension was mixed with 125 mL of alginate solution (4% *w/w*), completed with 5 mL of Polyethylenimine. After homogenization of the mixture, the suspension was dropped through a thin nozzle into an ionotropic gelation bath, constituted of 1 L of CaCl_2_ solution (1% *w/w*) containing 5 mL of glutaraldehyde (GA, 50% *w/w*). The alginate fraction (from algal biomass and pure biopolymer) was ionotropically gelled with calcium while glutaraldehyde reacts with amine groups on PEI to form a supplementary crosslinking network. The beads remained overnight in the solution before being thoroughly washed with demineralized water. The beads (APEI) were finally freeze-dried (−52 °C, 0.1 mbar, for two days). 

Prior to functionalization of APEI beads, the stability of the beads was chemically strengthened by complementary crosslinking using poly(ethyleneglycol) diglycidyl ether (3 mL dissolved into 90 mL of isopropanol): 5 g of APEI beads were mixed under reflux with the cross-linking solution. Cross-linked beads were finally recovered by filtration and successively rinsed with demineralized water and methanol, before being vacuum dried at 50 °C overnight. 

**Scheme 2:**
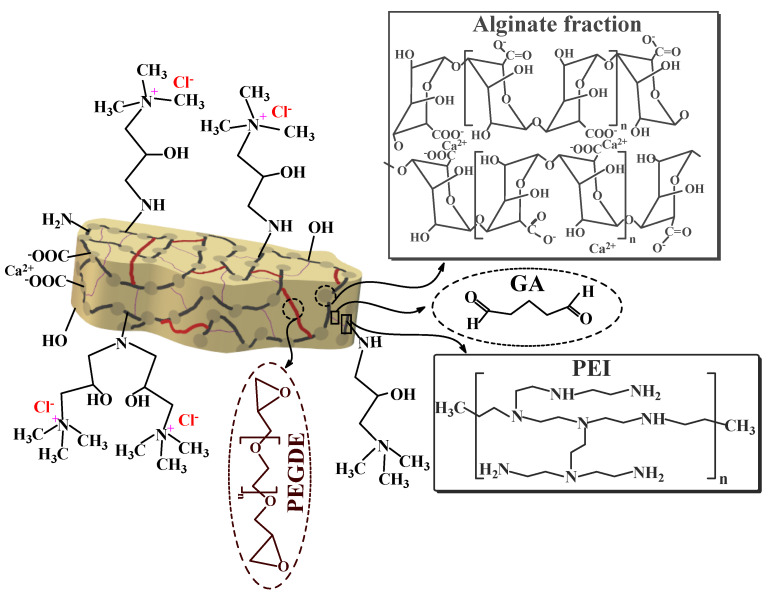
Structure of the sorbent.

Five g of cross-linked beads were dispersed into 140 mL DMF:H_2_O (1:1, *v*/*v*) mixture in a three-necked reactor. Glycidyltrimethylammonium chloride (10 g) was added to the suspension under reflux (T: 73 °C for 24 h) with gentle agitation. Produced quaternized beads (Q-APEI) were washed three times with hot water and methanol before being vacuum dried overnight at 50 °C. Scheme 2 shows the chemical structure of the sorbent, while Scheme S1 (see Supplementary Information) summarizes the main steps of the functionalization of APEI beads.

### 4.3. Material Characterization

Conventional analytical methods were used for characterizing the materials (APEI and Q-APEI) for elemental analysis, textural properties (BET analysis), thermogravimetric analysis (TGA analysis), and morphological observation (SEM) and SEM–EDX, (XL30-ESEM, Philips, FEI, Thermo Fisher Scientific, Hillsboro, OR, USA). These physical and chemical properties have been previously reported [41]. Briefly, Q-APEI is characterized by a specific surface area close to 34 m^2^ g^−1^, with a porous volume of 0.181 cm^3^ g^−1^ and an average pore size of 183–230 nm. The nitrogen content in Q-APEI represents around 8.57% *w/w*, or 6.12 mmol N g^−1^. The thermogravimetric analysis showed several steps of degradation corresponding to water desorption, degradation of amine compounds, depolymerization, and char degradation. The residual fraction does not exceed a few percent at 800 °C. The sorbent is roughly stable until temperature reaches around 230 °C.

FT-IR spectra were obtained using an IRTracer-100 FT-IR spectrometer (Shimadzu, Tokyo, Japan). All the samples were dried at 60 °C before being analyzed. Samples were conditioned as KBr disk. XPS spectra were collected using an ESCALAB 250XI+ instrument (Thermo Fischer Scientific, Inc., Waltham, MA, USA) with monochromatic X-ray Al Kα radiation (1486.6 eV). The signals corresponding to Ag *3*d_5/2_ (ΔBE: 0.45 eV) and C *1*s (ΔBE: 0.82 eV) were used for calibrating the analytical procedure. The full-spectrum pass energy and narrow-spectrum pass energy were set at 50 and 20 eV, respectively. SEM (scanning electron microscopy) observations were performed on a Phenom ProX SEM, Thermo Fisher Scientific, Eindhoven, Netherlands) at an accelerating voltage of 15 kV. The chemical composition of the samples was characterized by energy dispersive X-ray analysis (integrated to Phenom ProX SEM).

The pH-drift method was used for the determination of pH_PZC_ (pH of zero charge) [87]. Two series of 0.1 and 1 M NaCl solutions were prepared (initial pH varying between 1 and 11) and mixed with the sorbent for 48 h (sorbent dosage: 2 g L^−1^). The equilibrium pH (pH_eq_) was measured in the filtrate using a S220 Seven Compact pH/ Ionometer (Mettler-Toledo Instruments, Shanghai, China). The pH_PZC_ was defined by the pH value that corresponds to pH_0_ = pH_eq_.

### 4.4. Sorption Studies

#### 4.4.1. Sorption and Desorption Tests

Sorption studies were carried out in the batch mode (temperature: 22 ± 2 °C; agitation speed: 170 rpm). A given amount of sorbent (m, g) was mixed with a fixed volume (V, L) of metal-containing solution (C_0_, mmol U L^−1^) at fixed initial pH (pH_0_). Samples are collected and filtrated at fixed contact times for studying uptake kinetics; for equilibrium tests, the contact time was set to 48 h. For sorption isotherms, the initial concentration was varied between 10 and 500 mg U L^−1^ (i.e., 0.043–2.13 mmol U L^−1^). The pH was not adjusted during sorption tests but the equilibrium pH was systematically monitored. Residual metal concentration (C_eq_, mmol U L^−1^) was determined by inductively-coupled plasma atomic emission spectrometry (ICP-AES, ICPS-7510, Shimadzu, Tokyo, Japan), after filtered on 1.2-µm pore size filter membranes. The sorption efficiency was calculated as well as the sorption capacity (q_eq_, mmol U g^−1^) deduced from the mass balance equation: q_eq_ = (C_0_ − C_eq_) × V/m. For experiments involving different metal ions, the same sorption procedure was adopted. The metal concentration was determined by UV-Visible spectroscopy (Shimadzu UV-160A, Tokyo, Japan) for U(VI) and REEs using 0.05% *w/w* Arsenazo III colorimetric method at 655 and 654 nm, respectively [88]. Zinc and copper ions were analyzed by atomic absorption spectrometry (Unicam 969, Thermo Electron Corporation).

An acidic NaCl solution (0.5 M NaCl/0.5 M HCl) was used for processing desorption of uranyl from metal-loaded sorbent. For desorption kinetics, the beads loaded with uranyl from the study of uptake kinetics were used. The same batch procedure (as for sorption study) was used for testing the regeneration of the sorbent through a series of five cycles of sorption and desorption.

Details of experimental procedures are systematically reported in the caption of the figures (sorbent dosage, SD, g L^−1^; metal concentrations, pH_0_ and pH_eq_, temperature, agitation time and agitation speed, etc.). Sorption tests are duplicated (Figures show this duplication comparing Series #1 and Series #2).

#### 4.4.2. Modeling

Tables S1 and S2 (see Additional Material Section) report the conventional equations used for modeling uptake kinetics (pseudo-first and pseudo-second order rate equations, PFORE and PSORE; the Crank equation for simulating the resistance to intraparticle diffusion) and sorption isotherms (Langmuir, Freundlich, and Sips equations). The parameters of the models were obtained using non-linear regression analysis (facilities from Mathematica ^®^ software); the fit adjustment was measured calculating the determination coefficients (comparing experimental data and simulated data) and also comparing calculated and experimental sorption capacities (maximum sorption capacities for sorption isotherms and equilibrium sorption capacities for uptake kinetics).

### 4.5. Processing of Uranium-Bearing Ore and Uranium Recovery

Sorption tests on industrial effluents were carried out on the leachates of ore samples collected in the so-called Alloga locality (close to Abu Zeneima, in SW Sinai, Egypt). The ore is characterized as a polymetallic carbonaceous shale. Figure S12 shows the geological map corresponding to Abu Zeneima area. 

The mineralogical analysis showed the presence of several economic minerals including monazite (phosphate of rare earth elements), sklodowskite (Mg(UO_2_)_2_(HSiO_4_)_2_·5H_2_O), azurite (Cu_3_(CO_3_)_2_(OH)_2_), malachite (Cu_2_CO_3_(OH)_2_), atacamite (Cu_2_Cl(OH)_3_), carrollite (Cu_2_Cl(OH)_3_), chalcopyrite (CuFeS_2_), siegenite ((Ni,Co)_3_S_4_), polydymite (Ni_3_S_4_), violarite (Fe^2+^Ni_2_^3+^S_4_), and zircon (ZrSiO_4_). 

Major oxide and trace elements were analyzed by conventional methods (including fusion, mineralization and metal analysis by atomic absorption spectrometry [6]). A wide range of valuable metals are present in the polymetallic ore, including Cu (2.4% *w/w*), REEs (0.22%), U (0.62%), V (0.13%), Ni (0.25%), and Co (0.11%) in addition to a remarkable amount of Zr (0.025%) (from [89]). 

A series of leaching studies were carried out for the optimization of sulfuric acid leaching (stirred reactor), alkali agitation leaching, and pug leaching method. The pug leaching process achieved the highest efficiency in metal release under the following conditions: 1.35 ton of H_2_SO_4_ was stirred with 1 ton of ore at T: 110 °C for 2 h. leachates contained: 6.5 g Fe L^−1^, 3.4 g Cu L^−1^, 0.22 g REEs L^−1^, 0.6 g U L^−1^, and 0.19 g V L^−1^.

The pregnant leach solution (PLS) was first treated by cementation (using Fe powder, 0.3 g of iron for 100 mL of PLS) to precipitate copper as Cu metal. The reaction was carried out at pH 0.7, for 30 min at T: 70 °C. 

The Cu-depleted leachate was then treated with oxalic acid for selectively separating rare earths from uranium in the liquor. A 10% *w/w* oxalic acid solution was added to the liquor at pH 1.1, at T: 22 °C, under agitation for 45 min. Turbid RE oxalate suspension was recovered by filtration. Prior treating the residual solution by sorption on Q-APEI, the pH was controlled to 5 to remove the major part of iron by precipitation. The filtrate was separated into two stock solutions that were controlled at pH 2 and pH 4, respectively. 

Sorption tests on these solutions were carried out in stirred tank reactor by contact of 200 mg of sorbent with 50 mL of pre-treated leachates. Samples were collected at fixed contact times (i.e., 1, 2, 4, 10, 20, and 24 h) for quantification of residual concentrations (U, Cu, and Fe) in filtrated solutions. At the end of sorption tests, uranium was desorbed from loaded resins using 0.5 M HCl solution (V: 30 mL) under agitation for 30 min. The yellow cake was obtained by precipitation of the eluate with 1 M NaOH solution, adjusting the pH to 9. The semi-quantitative composition of the yellow cake was determined by EDX analysis.

## Figures and Tables

**Table 1:** Parameters of models for U(VI) uptake kinetics.

Model	Parameter	Fitted Value First Series	Fitted Value Second Series
Experimental	q_eq_ (mmol U g^−1^)	0.343	0.349
PFORE	q_eq,1_ (mmol U g^−1^)	0.358	0.365
	k_1_ × 10^2^ (min^−1^)	9.19	9.61
	R^2^	0.992	0.989
PSORE	q_eq,2_ (mmol U g^−1^)	0.451	0.455
	k_2_ × 10^2^ (L mmol^−1^ min^−1^)	20.2	21.3
	R^2^	0.980	0.976
RIDE	D_e_ × 10^8^ (m^2^ min^−1^)	2.59	3.28
	R^2^	0.969	0.967

**Table 2:** Parameters of models for U(VI) sorption isotherms.

Model	Parameter	APEI	Q-APEI
Experimental	q_m_ (mmol U g^−1^)	0.127	0.855
Langmuir	q_m,L_ (mmol U g^−1^)	0.150	0.938
	b_L_ (L mmol^−1^)	3.16	5.06
	R^2^	0.988	0.976
Freundlich	k_F_	0.106	0.754
	n_F_	2.67	2.99
	R^2^	0.915	0.969
Sips	q_m,S_ (mmol U g^−1^)	0.140	1.297
	b_S_ (L mmol^−1^)	4.77	1.47
	n_S_	0.834	1.65
	R^2^	0.986	0.984

**Table 3:** Comparison of sorbents for U(VI) recovery in acidic solutions (sorption performances: optimum pH, equilibrium time, and Langmuir constants).

Sorbent	pH	t_eq_ (min)	q_m,L_ (mmol U g^−1^)	b_L_ (L mmol^−1^)	Ref.
*Sargassum*	4	180	2.40	0.170	[71]
Algal/yeast/SiO_2_	4	180	0.210	7.14	[72]
Grapefruit peel	5	90	0.592	7.45	[73]
Rice husk	4	320	0.190	23.6	[74]
*Landoltia punctata*	4-5	1440	0.305	2.36	[75]
Eucalytus wood biochar	5.5	20	0.114	5.95	[76]
Sugar beet pulp	8	120	0.086	6.43	[77]
Amidoximated marine mycelium	5	120	1.56	0.378	[70]
Tulsion CH-96	3–4 M HNO_3_	600	0.294	1.48	[78]
Amberlite CG-400	3.5	360	0.472	21.9	[79]
Phosphorus PStyr/DVB	5	240	0.378	8.33	[18]
DETA-magnetic chitosan	3.5	120	0.274	295	[80]
Carminic acid impregnated resin	5	120	0.798	29.2	[11]
D2EHPA-impregnated polymer beads	4	180	0.079	5.24	[12]
Amberlite IRA-402	3	90	0.895	11.9	[81]
Picolylamine funct. resin	5.3	120	2.31	164	[68]
Amidoximated MCM-41 SiO_2_	5	40	1.86	8.5	[69]
Amidoximated MCM-41 SiO_2_	5	90	1.62	225	[31]
Phosphonate/MoS_2_	5.5	360	0.949	9.52	[82]
Porous hydroxyapatite	3.0	30	0.468	1113	[83]
Carboxylated-Zn-MOF	4	60	0.544	11.2	[84]
Amidoxime funct. catechol iron oxide NPs	6.5	180	0.256	9.52	[85]
APEI	4	-	0.150	3.16	*This work*
Q-APEI	4	40	0.938	5.06	*This work*

**Table 4:** Modeling of kinetic profiles for U(VI) desorption from loaded Q-APEI sorbent—PFORE and PSORE model [86].

Sorbent	Model	PFORE		PSORE		
	Parameter	k_D1_ (min^−1^)	R^2^	β_2_	k_D2_ (min^−1^)	R^2^
Q-APEI 1st Series		0.077	0.957	0.950	0.136	0.967
Q-APEI 2nd series		0.086	0.917	0.950	0.157	0.914

PFORE: $\frac{q\left(t\right)}{q_{0}}=e^{-k_{D1}t}$ with: k_D1_ the apparent rate coefficient for desorption (min^−1^) PSORE: $\frac{q\left(t\right)}{q_{0}}=\frac{1}{\beta_{2}+k_{D2}t}$ with: k_D2_ the apparent rate coefficient for desorption (min^−1^) and β_2_ (dimensionless) the constant for PSORE (in desorption).

**Table 5:** U(VI) desorption and sorbent recycling for Q-APEI.

Cycle	Sorption Efficiency (%)		Desorption Efficiency (%)	
	Average	S.D.	Average	S.D.
1	98.5	0.2	100.1	0.2
2	98.0	0.1	99.9	0.9
3	98.0	0.1	98.9	0.2
4	97.9	0.0	99.4	1.0
5	97.8	0.2	98.4	0.3

(Experimental conditions: Sorption: C_0_: 98 mg U L^−1^ = 0.413 mmol U L^−1^; pH: 4; SD: 1 g L^−1^; time: 24 h; T: 22 ± 2 °C; agitation speed: 170 rpm/Desorption: eluent: 0.5 M CaCl_2_/0.5 M HCl; SD: 2.5 g L^−1^; time: 2 h T: N; agitation speed: 170 rpm).

## Supplementary Materials

The following are available online at https://www.mdpi.com/2310-2861/6/2/12/s1, Scheme S1: Main steps of the procedure of quaternization of APEI beads. Table S1: FTIR assignments peaks and corresponding wavenumbers (cm^−1^) of APEI, Q-APEI, Q-APEI+U, and Q-APEI after desorption and Q-APEI after 5 cycles of sorption/desorption. Table S2: H-Res. XPS peaks of C *1*s, O *1*s, N *1*s, Ca *2*p and S *2*p for APEI and Q-APEI sorbent. Table S3: Assignments, Binding energies (BEs), Full width at half-maximum (FWHM) and Atomic Fractions (AF) (%) of APEI, and Q-APEI sorbents. Table S4: H-RES. XPS characterization of Q-APEI after loading with uranyl ions. Table S5: Assignments, Binding energies (BEs), Full width at half-maximum (FWHM) and Atomic Fractions (AF) (%) of APEI, and Q-APEI sorbents. Table S6: SEM–EDX analysis of Q-APEI after U(VI) sorption at different pH values. Table S7: Uptake kinetics modeling—PFORE (pseudo-first order rate equation), PSORE (pseudo-second order rate equation), and RIDE (resistance to intraparticle diffusion equation—Crank equation). Table S8: Sorption isotherm modeling [90,91]. Table S9: Pre-treatment of PLS (Abu Zeneima ore): initial concentrations of major elements and residual concentrations after Cu cementation, and after separation of REEs (by oxalic precipitation) and iron abatement (pH control). Table S10: Semi-quantitative EDX analysis of loaded sorbent (pre-treated leachate at pH 2 and 4). Figure S1: Determination of the pH_PZC_ of APEI and Q-APEI sorbents by the pH-drift method (Sorbent dosage, SD: 2 g L^−1^; contact time: 48 h; agitation speed: 170 rpm; background salt NaCl 1 M (dashed lines) and 0.1 M (solid lines); T: 22 ± 2 °C). Figure S2: Effect of equilibrium pH on the distribution ratio of U(VI) (log_10_ plot) for APEI and Q-APEI sorbents (Sorbent dosage, SD: 0.333 g L^−1^; contact time: 48 h; agitation speed: 170 rpm; T: 22 ± 2 °C). Figure S3: U(VI) speciation in function of pH (a) and total metal concentration at pH 4 (b) (following the experimental conditions relevant to pH study and sorption isotherms, respectively; calculations performed using Visual Minteq, [92]). Figure S4: Variation of pH during U(VI) sorption using APEI and Q-APEI sorbents (Sorbent dosage, SD: 0.333 g L^−1^; contact time: 48 h; agitation speed: 170 rpm; T: 22 ± 2 °C). Figure S5: U(VI) uptake kinetics using Q-APEI sorbent—Modeling with the PSORE (a) and the RIDE (b) (SD: 0.3 g L^−1^; pH_0_: 4; pH_eq_: 3.79–3.71; C_0_: 0.214 mmol U L^−1^ and 0.249 mmol U L^−1^ for 1st and 2nd series, respectively; agitation speed: 170 rpm; T: 22 ± 2 °C). Figure S6: Semi-quantitative EDX analysis of copper-cake collected after cementation. Figure S7: Semi-quantitative EDX analysis of iron-cake after pH control at pH 5. Figure S8: Effect of time on uranium sorption from multi-component solutions (C_0_: 350–346 mg U L^−1^ = 1.47–1.45 mmol U L^−1^; T: 22 ± 2 °C; SD: 0.125 g L^−1^; agitation speed: 170 rpm). Figure S9: Distribution ratios of Cu, Fe, and U after sorption on Q-APEI at pH_eq_: 2.98 and 3.81 (pH_0_: 2 and 4, respectively) from pre-treated PLS (inserted numbers represent the relevant sorption capacities at equilibrium, µmol g^−1^). Figure S10: Selectively coefficients SC_U/Cu_ and SC_U/Fe_ for metal sorption from pre-treated PLS (C_0_: 3 mg Cu L^−1^ = 0.047 mmol Cu L^−1^, 1925 mg Fe L^−1^ = 34.47 mmol Fe L^−1^, 350-346 mg U L^−1^ = 1.47-1.45 mmol U L^−1^; SD: 0.125 g L^−1^; agitation speed: 170 rpm; agitation time: 24 h). Figure S11. Semi-quantitative analysis of the yellow cake produced from the eluates of sorbent after U-loading from ore leachates at pH 2 and pH 4. Figure S12: Alloga locality of Abu Zienema area, South Western Sinai, Egypt.
