# Supplementary material for: Quaternization of Composite Algal/PEI Beads for Enhanced Uranium Sorption—Application to Ore Acidic Leachate
Source: Gels. 2020 Mar 30;6(2):12. doi: 10.3390/gels6020012 (PMC7345210; doi:10.3390/gels6020012)
Supplement: Supplementary file 1 [file gels-06-00012-s001.pdf]

gels

Quaternization of composite algal/PEI beads for enhanced uranium sorption – Application to ore acidic leachate

Mohammed F. Hamza [1,2], Amal E. Mubark [2], Yuezhou Wei [1,3,*], Thierry Vincent [4] and Eric Guibal [4,*]

[1] Guangxi Key Laboratory of Processing for Non-ferrous Metals and Featured Materials, School of Resources, Environment and Materials, Guangxi University, Nanning 530004, PR China.; m_fouda21@hotmail.com, yzwei@gxu.edu.cn
[2] Nuclear Materials Authority, POB 530, El-Maadi, Cairo, Egypt; m_fouda21@hotmail.com, amal.mubark@yahoo.com
[3] Shanghai Jiao Tong University, Shanghai 200240, China; yzwei@gxu.edu.cn
[4] Polymers Composites and hybrids (PCH), IMT-Mines Ales, 6, avenue de Clavières, F-30319 Alès cedex, France; thierry.vincent@mines-ales.fr, eric.guibal@mines-ales.fr
* Correspondence: eric.guibal@mines-ales.fr, +33 (0)466782734 (E.G.) and yzwei@gxu.edu.cn, +86-771-3224990 (Y.W.)

ADDITIONAL MATERIAL SECTION

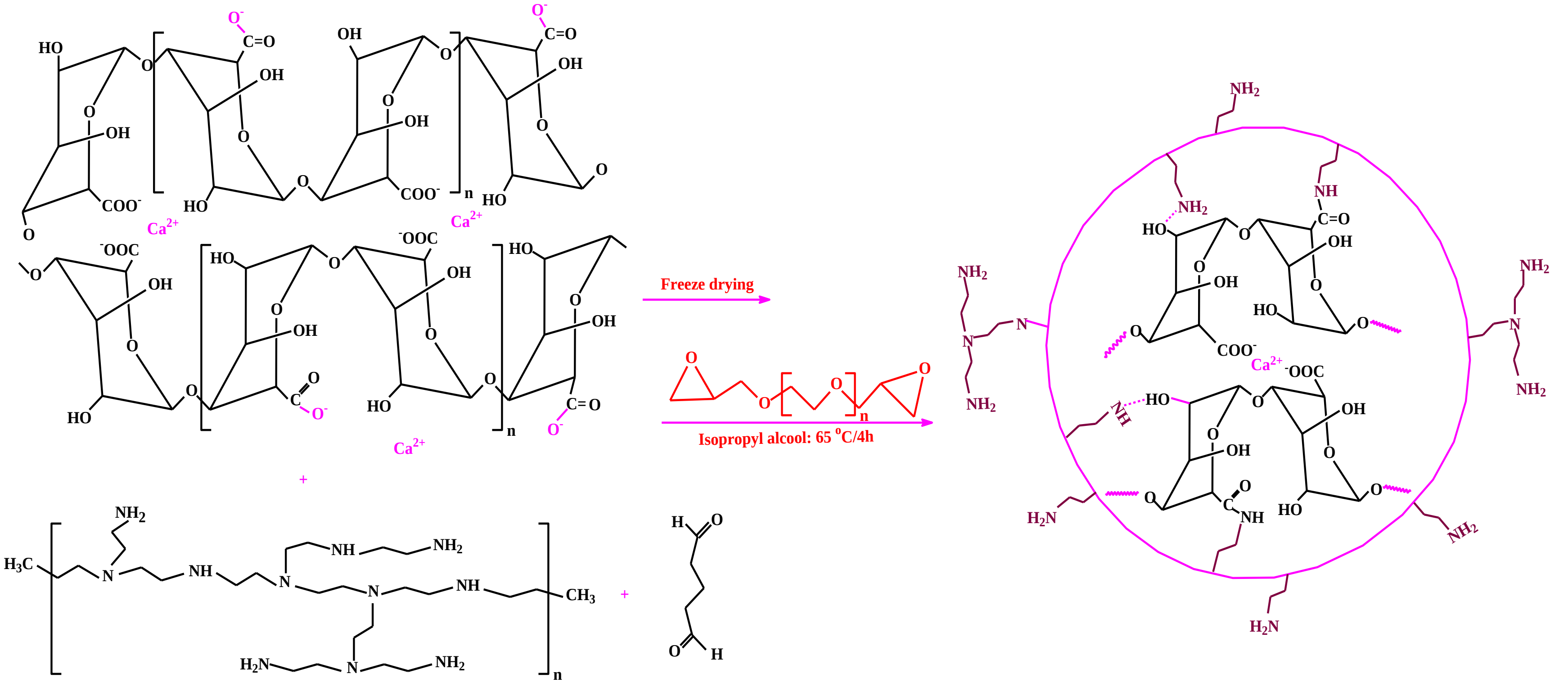
Freeze drying
Isopropyl alcool: 65 °C/4h

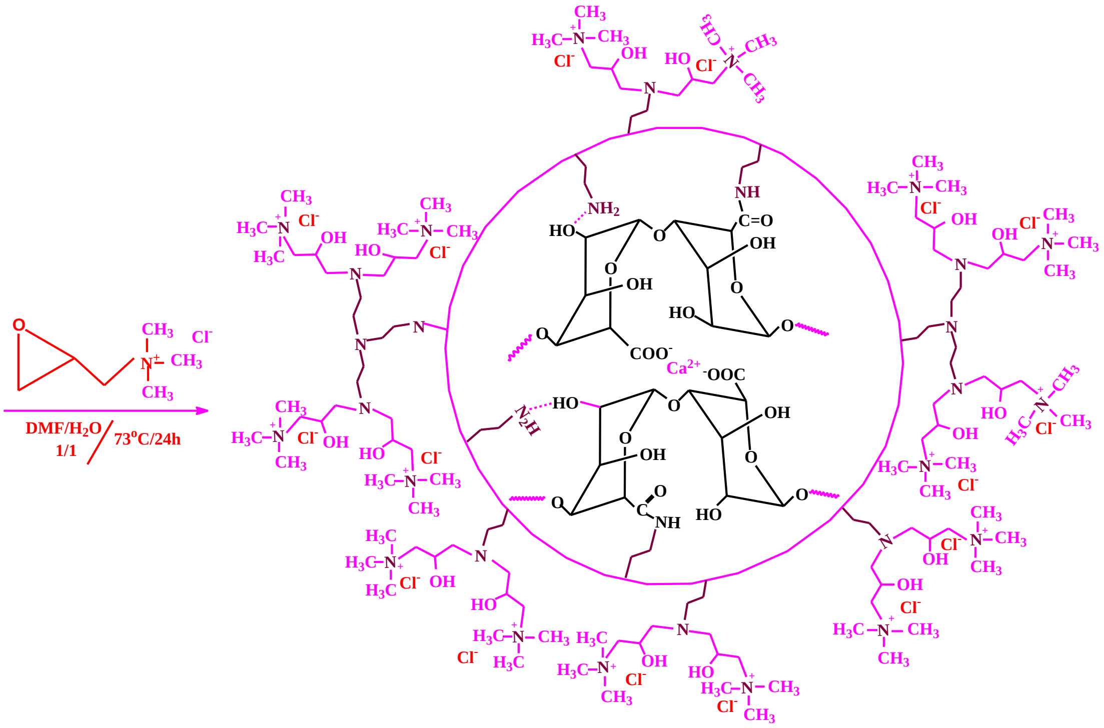

Scheme S1. Main steps of the procedure of quaternization of APEI beads.

Table S1. FTIR assignments peaks and corresponding wavenumbers (cm^{-1}) of APEI, Q-APEI, Q-APEI+U, Q-APEI after desorption and Q-APEI after 5 cycles of sorption/desorption.

Vibration	Ref.	Wn. in ref.	APEI	Q-APEI.	Q-APEI + U	After U des.	After 5 cycles
O-H overlapped with N-H stretching	[1, 2]	3500 -3000	3445	3447	3136<br>3441	3217	3211
Open-chain imino (-C=N-)	[3, 4]	1690–1590	1632	1618	1641<br>1730	1624	1622
N-H bend primary/secondary amine overlapped with carboxylic acid salt	[3]	1650–1550					
C-H bend asym. /methylene groups linked to N^+	[3, 5-7]	1485–1430	**	1468	1385	1396	1396
Carboxylate (carboxylic acid salt)	[3]	1420–1300		1411			
O-H bend of primary/ secondary in-plane	[3]	1350–1260	1400	1313			
C-O stretch primary/secondary alcohol	[3]	1100-1050	1122	1094	1105	1097	1099
C-N stretch of amine	[3, 6, 8-10]	1210–1020	1032	1030	1032	1033<br>916	1032<br>916
Methylene -(CH_2)*n*- rocking	[3]	750–720	816	804	841	839	825
Alcohol, OH out-of-plane bend		720–590			794		
Sulfate ion		680–610			617 (strong)		625 (weak)

Table S2. H-RES. XPS peaks of C *1*s, O *1*s, N *1*s, Ca *2*p *and* S *2*p for APEI and Q-APEI sorbent.

**Signal**	**APEI**	**Q-APEI**
C *1*s	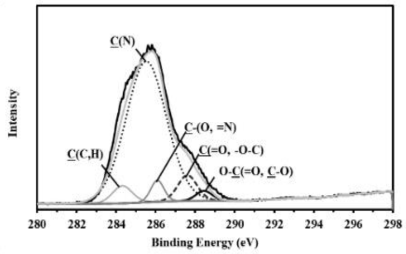	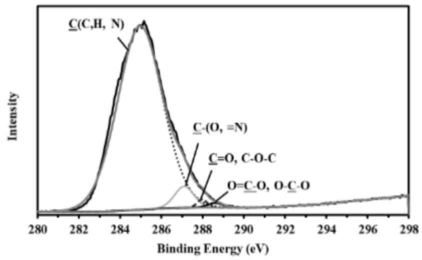
O *1*s	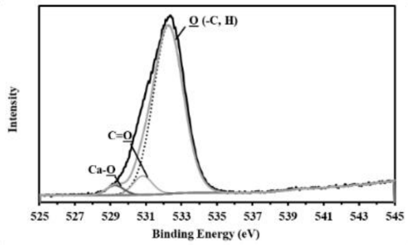	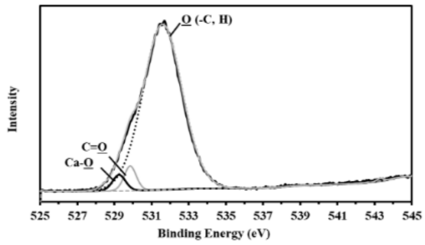
N *1*s	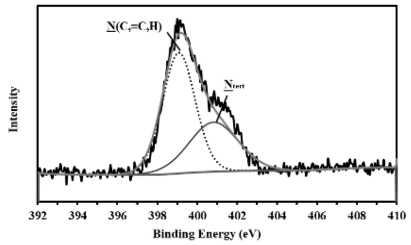	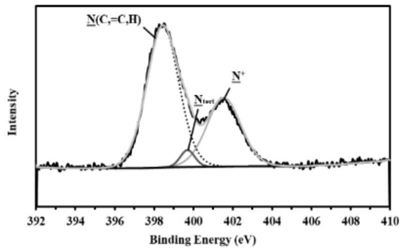
Ca *2*p	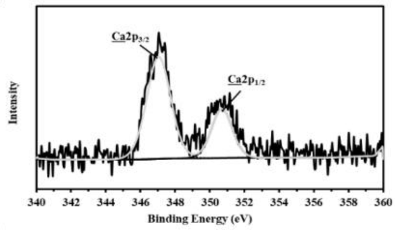	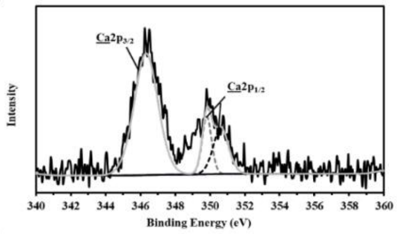
S *2*p	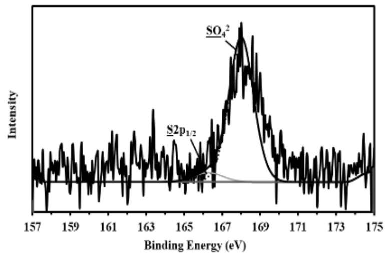	

Table S3. Assignments, Binding energies (BEs), Full width at half-maximum (FWHM) and Atomic Fractions (AF, %) of APEI, and Q-APEI sorbents.

Signal	Assignments	APEI			Q-APEI		
		BE (eV)	FWHM	AF (%)	BE (eV)	FWHM	AF (%)
C *1*s	C (C, H, N)	284.27	0.77	4.94	284.97	2.72	94.27
	C(-NH, NH_2)	285.41	2.44	84.6			
	C(-O, =N)	286.08	1.82	2.71	286.86	1.56	5.01
	C(=O, -O-C)	287.6	1.15	5.7	287.92	0.56	0.43
	O-C(=O, C-O)	288.44	0.71	2.05	288.58	0.6	0.29
O *1*s	O-Ca	529.29	1.17	4.43	529.22	0.83	3.54
	C=O	530.7	1.27	12.82	530.19	0.77	4.72
	O(C, H)	532.27	2.08	82.75	531.89	2.47	91.74
N *1*s	N(C,=C, H)	399.2	1.8	61.76	398.44	1.91	63.75
	$N_{tert.}$	400.74	1.5	38.24	399.7	0.87	3.44
	N^+				401.65	2.04	32.81
S 2p	S $2p_{1/2}$	165.98	1.82	19.03			
	SO_4^{2-}	168.01	1.51	80.97			
Cl 2*p*	Cl $2p_{3/2}$				196.42	1.56	60.65
	Cl $2p_{1/2}$				198.39	1.25	39.35
Ca 2p	Ca $2p_{3/2}$	347.12	1.62	64.41	346.36	1.6	66.87
	Ca $2p_{1/2}$	350.69	0.52	35.59	349.8<br>350.75	0.52<br>1.04	13.97<br>19.16

Table S4. H-RES. XPS characterization of Q-APEI after loading with uranyl ions.

**Signal**	**Q-APEI-U**	**Signal**	**Q-APEI-U**
C *1*s	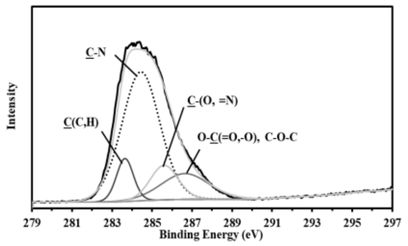	U *4*f	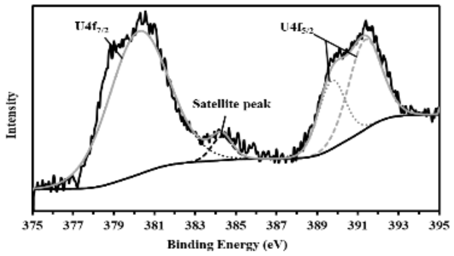
O *1*s	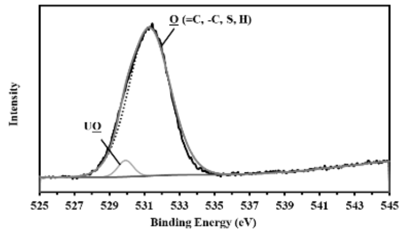	U 4d	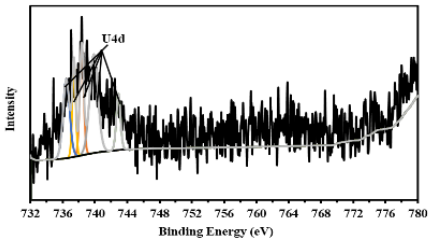
N *1*s	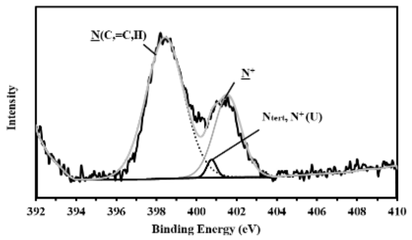	U 4p1	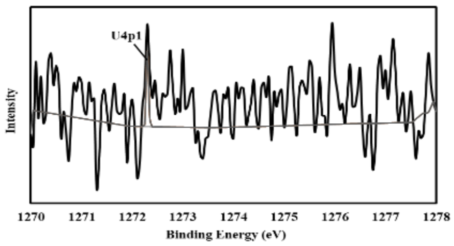
Ca 2p	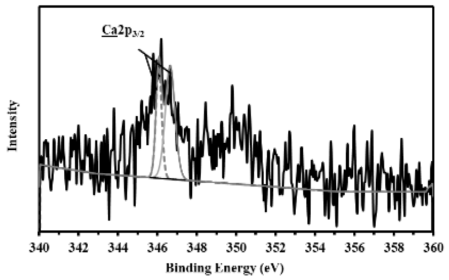	U 4p1	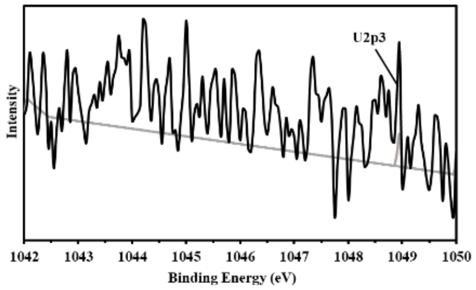
S 2p	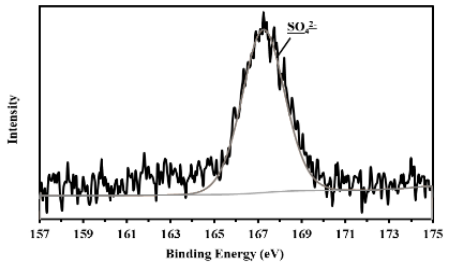		
Cl 2p			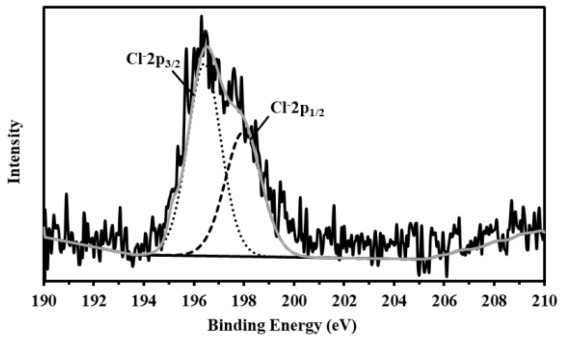

Table S5. Assignments, Binding energies (BEs), Full width at half-maximum (FWHM) and Atomic Fractions (AF, %) of APEI, and Q-APEI sorbents.

Signal	Assignments	BE (eV)	FWHM	AF (%)
C *1*s	C (C, H,N)	283.9	0.4	13.26
	C(-NH or NH_2)	284.58	2.88	59.01
	C(-O, =N)	285.85	0.52	12.16
	C(=O, -O-C), O-C-O	286.97	2.81	15.57
O *1*s	O(=C, C,H)	531.09	2.79	96.05
	U-O	529.92	1.04	3.95
N *1*s	N(C,=C, H)	398.43	2.23	69.15
	$N_{tert.}$, N-U	400.75	0.58	2.29
	N^+	401.55	1.6	28.56
S 2p	SO_4^{2-}	167.25	2.28	100
Ca 2p	Ca $2p_{3/2}$	346.07	0.34	37.03
		346.65	0.58	62.97
U 4f	U $4f_{7/2}$	380.23	3.48	61.74
	U $4f_{5/2}$	389.71	1.57	13.37
		391.34	1.9	21.31
	Satellite peak	384.31	1.22	3.58
U 4d	U 4d	738.35	0.57	18.83
		739.85	1.19	35.16
		737.4	0.48	13.33
		736.45	0.96	22.61
		742.9	0.6	10.07
U 4p1	U 4p1	1272.31	0.05	100
U 4p3	U 4p3	1048.93	0	100

Table S6. SEM-EDX analysis of Q-APEI after U(VI) sorption at different pH values.

pH	SEM	EDX
1	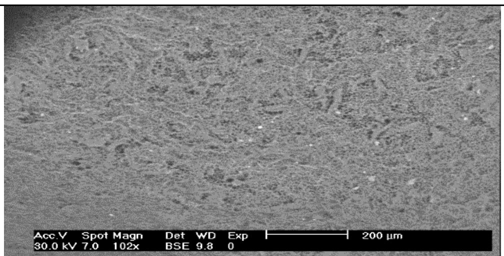	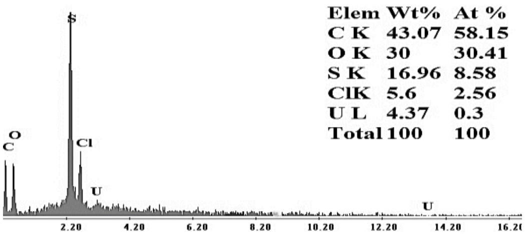
2	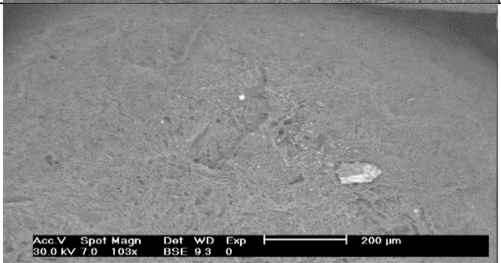	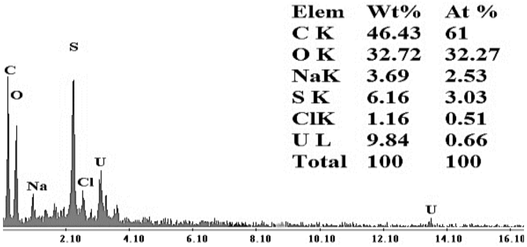
3	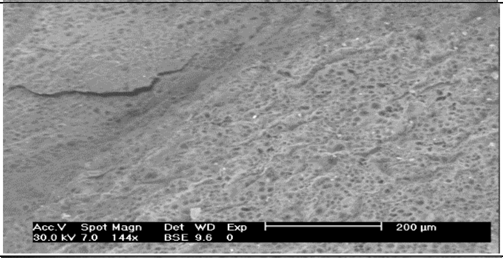	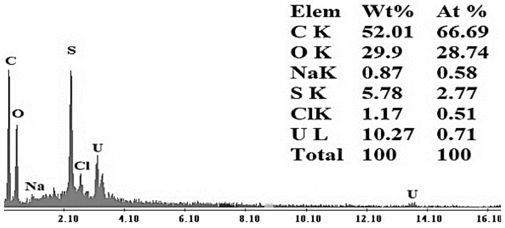
4	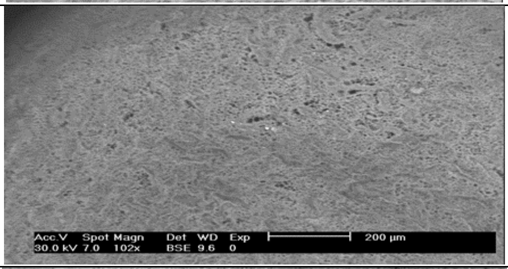	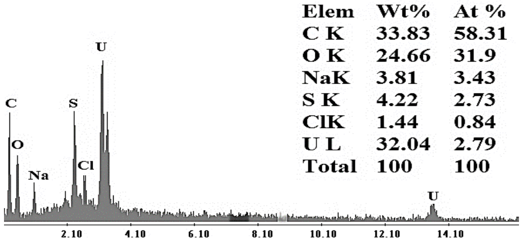
5	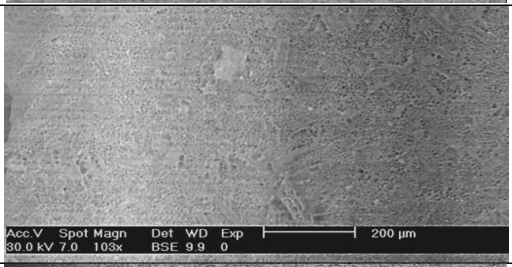	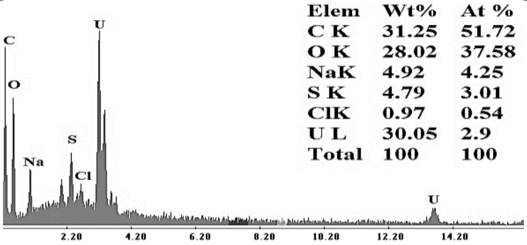
6	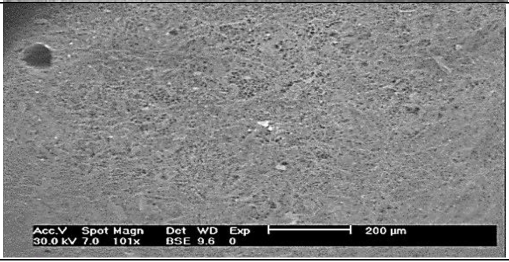	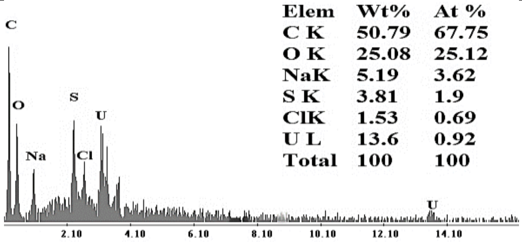

Table S7. Uptake kinetics modeling – PFORE (pseudo-first order rate equation), PSORE (pseudo-second order rate equation) and RIDE (resistance to intraparticle diffusion equation – Crank equation).

Model	Equation	Parameters	
PFORE [11]	$q(t) = q_{eq,1}(1 - e^{k_1 t})$	$q_{eq,1}$ (mg g^{-1})	k_1 (min^{-1})
PSORE [11]	$q(t) = \frac{q_{eq,2}^2 \times k_2 \times t}{1 + q_{eq,2} \times k_2 \times t}$	$q_{eq,2}$ (mg g^{-1})	k_2 (L mg^{-1} min^{-1})
RIDE [12]	$\frac{q(t)}{q_{eq}} = 1 - \sum_{n=1}^{\infty} \frac{6\alpha(\alpha+1)\exp\left(\frac{-D_e q_n^2 t}{r^2}\right)}{9 + 9\alpha + q_n^2 \alpha^2}$ With q_n being the non-zero roots of $\tan q_n = \frac{3 q_n}{3 + \alpha q_n^2}$ and $\frac{m q}{V C_o} = \frac{1}{1+\alpha}$	D_e (m^2 min^{-1})	

Table S8. Sorption isotherm modeling [13, 14]

Model	Langmuir	Freundlich	Sips
Equation	$q = \frac{q_{m,L} \times b_L \times C_{eq}}{1 + b_L \times C_{eq}}$	$q = k_F\ C_{eq}^{1/n}$	$q = \frac{q_{m,S} \times b_S \times {C_{eq}}_{eq}^{1/n_S}}{1 + b_S \times {C_{eq}}_{eq}^{1/n_S}}$
Parameters	$q_{m,L}$ (mg g^{-1})*	k_F ($mg^{1-1/n}$ g^{-1} $L^{-1/n}$)	$q_{m,S}$ (mmol g^{-1})*
	b_L (L mg^{-1})**	n (dimensionless)	b_S (L mg^{-1})**
	-	-	n_S (dimensionless)

*: Sorption capacity at saturation of the monolayer; **: Affinity coefficient.

Table S9. Pre-treatment of PLS (Abu Zeneima ore): initial concentrations of major elements and residual concentrations after Cu cementation, and after separation of REEs (by oxalic precipitation) and iron abatement (pH control).

Metal ion	Concentration (mg L^{-1})		
	PLS	After cementation	After REE (oxalic acid) and Fe (pH control) precipitation
Cu	34000	94.3	3
U	600	450	350
REE	220	214	5.3
Fe	6500	7700	1925

Table S10. Semi-quantitative EDX analysis of loaded sorbent (pre-treated leachate at pH 2 and 4).

<table>
<tr><th>pH</th><th>SEM micrograph</th><th>EDX analysis</th></tr>
<tr><td>2</td><td>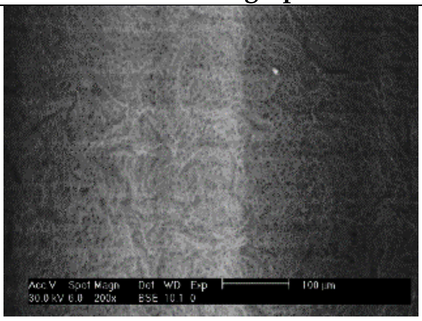
</td><td>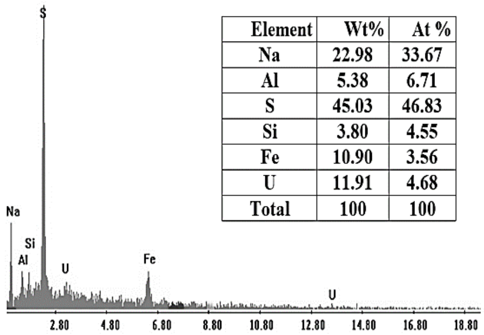
</td></tr>
<tr><td>4</td><td>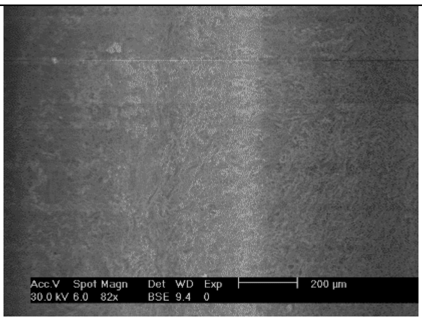
</td><td>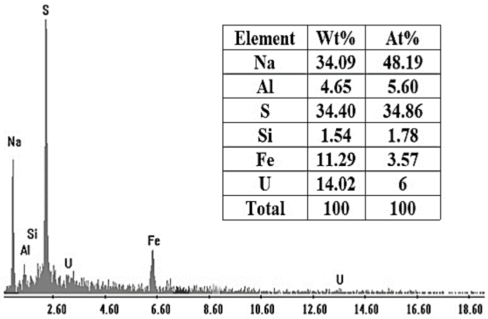
</td></tr>
</table>

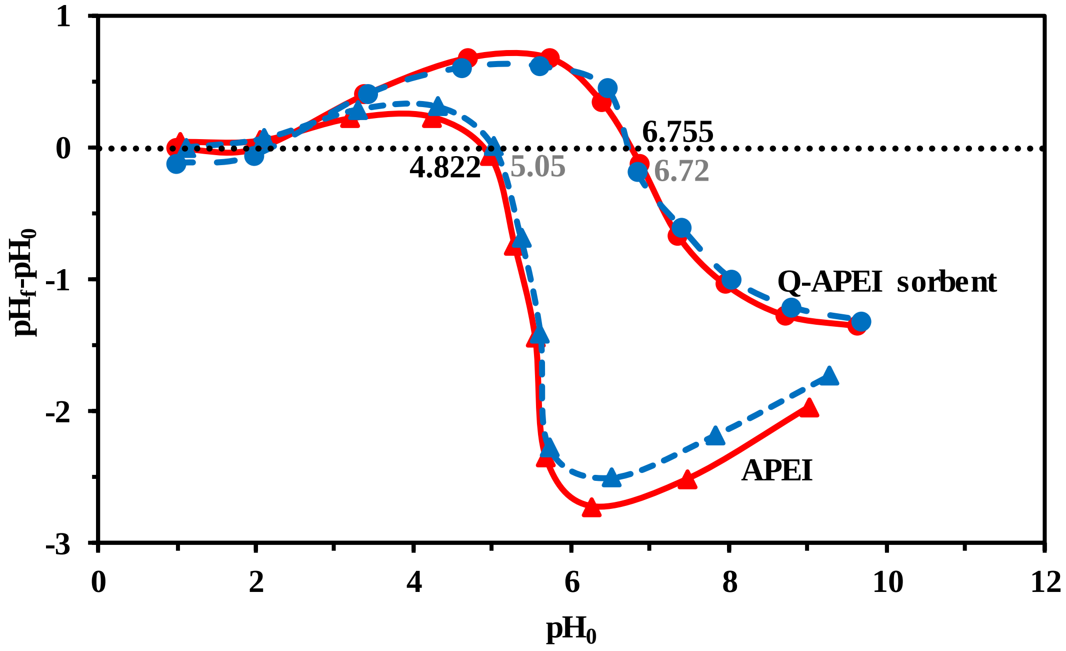

Figure S1. Determination of the pH_{PZC} of APEI and Q-APEI sorbents by the pH-drift method (Sorbent dosage, SD: 2 g L^{-1}; contact time: 48 h; agitation speed: 170 rpm; background salt NaCl 1 M (dashed lines) and 0.1 M (solid lines); T: 22 ± 2 °C).

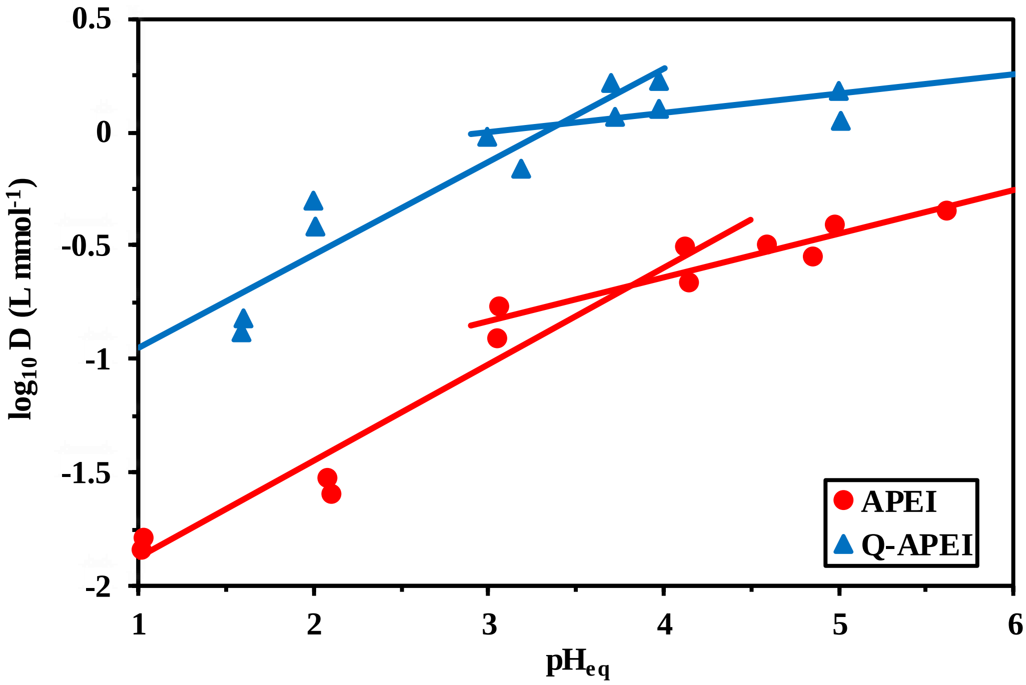

Figure S2. Effect of equilibrium pH on the distribution ratio of U(VI) (log_{10} plot) for APEI and Q-APEI sorbents (Sorbent dosage, SD: 0.333 g L^{-1}; contact time: 48 h; agitation speed: 170 rpm; T: 22 ± 2 °C).

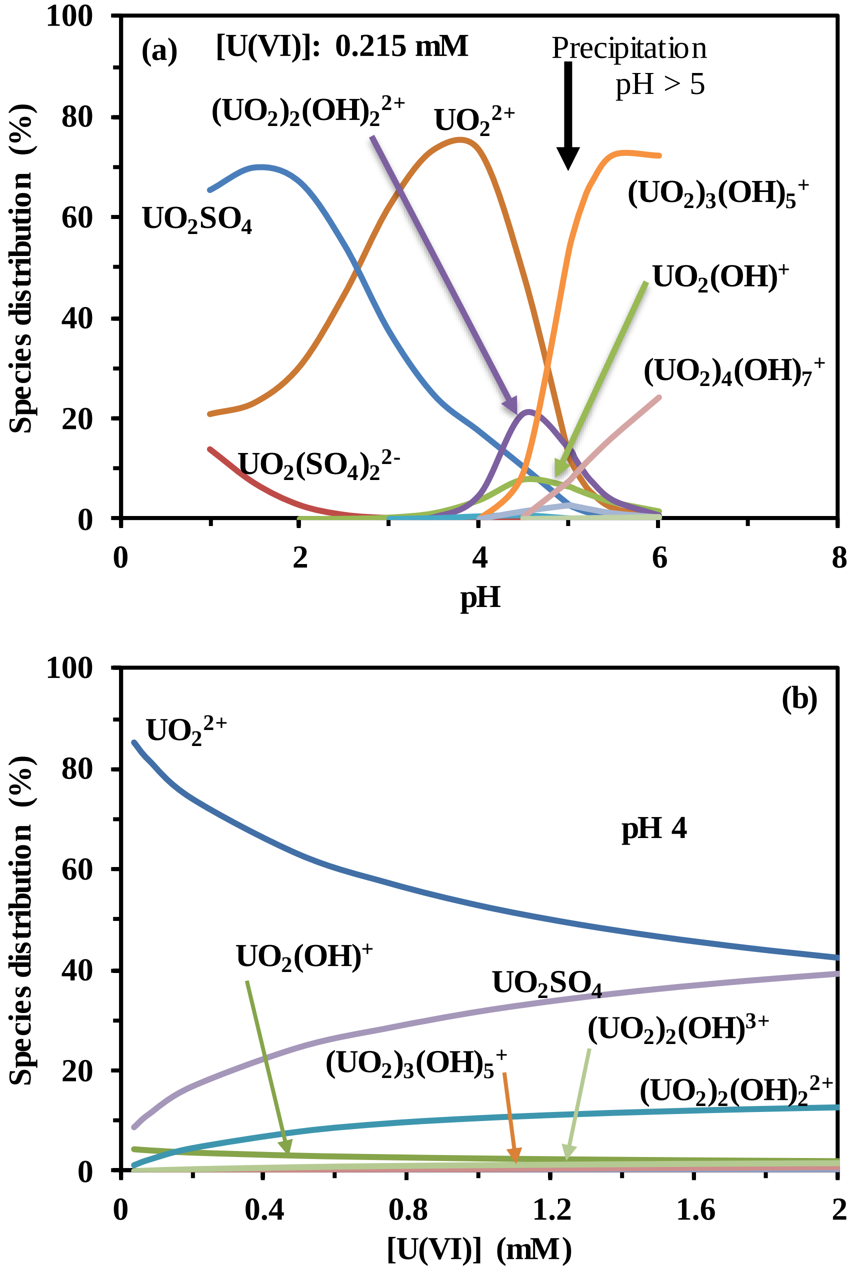

Figure S3. U(VI) speciation in function of pH (a) and total metal concentration at pH 4 (b) (following the experimental conditions relevant to pH study and sorption isotherms, respectively; calculations performed using Visual Minteq, [15]).

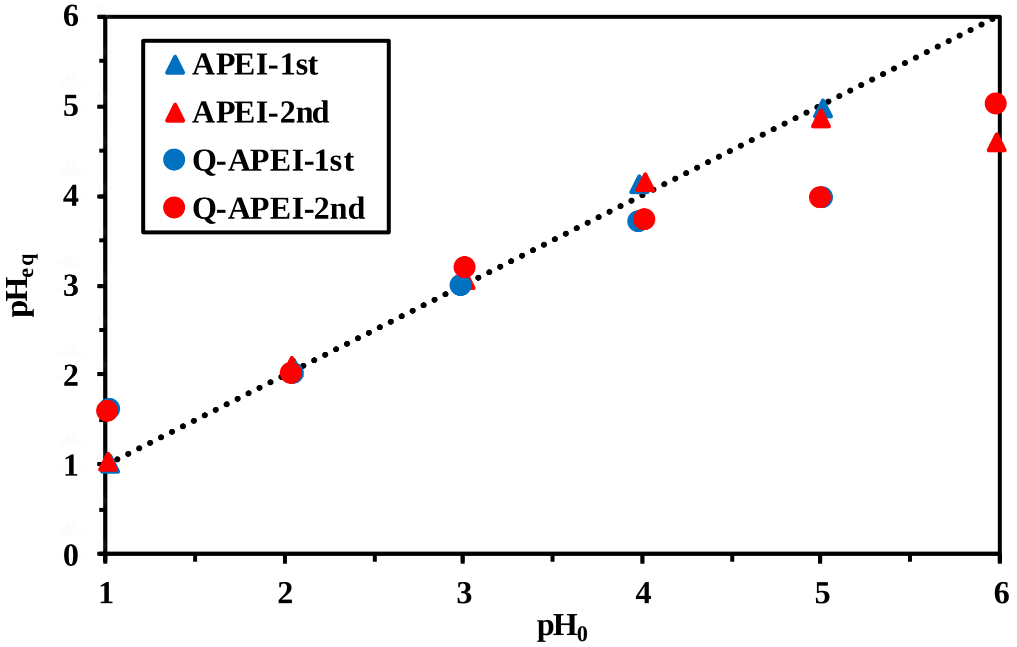

Figure S4. Variation of pH during U(VI) sorption using APEI and Q-APEI sorbents (Sorbent dosage, SD: 0.333 g L^{-1}; contact time: 48 h; agitation speed: 170 rpm; T: 22 ± 2 °C).

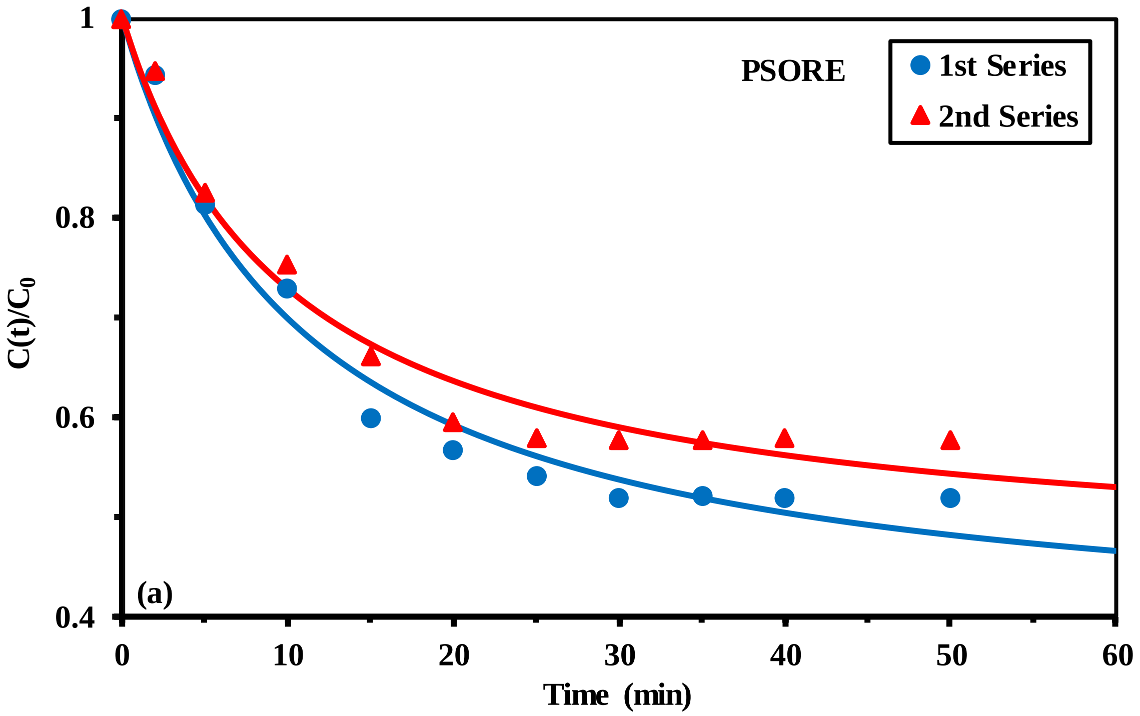

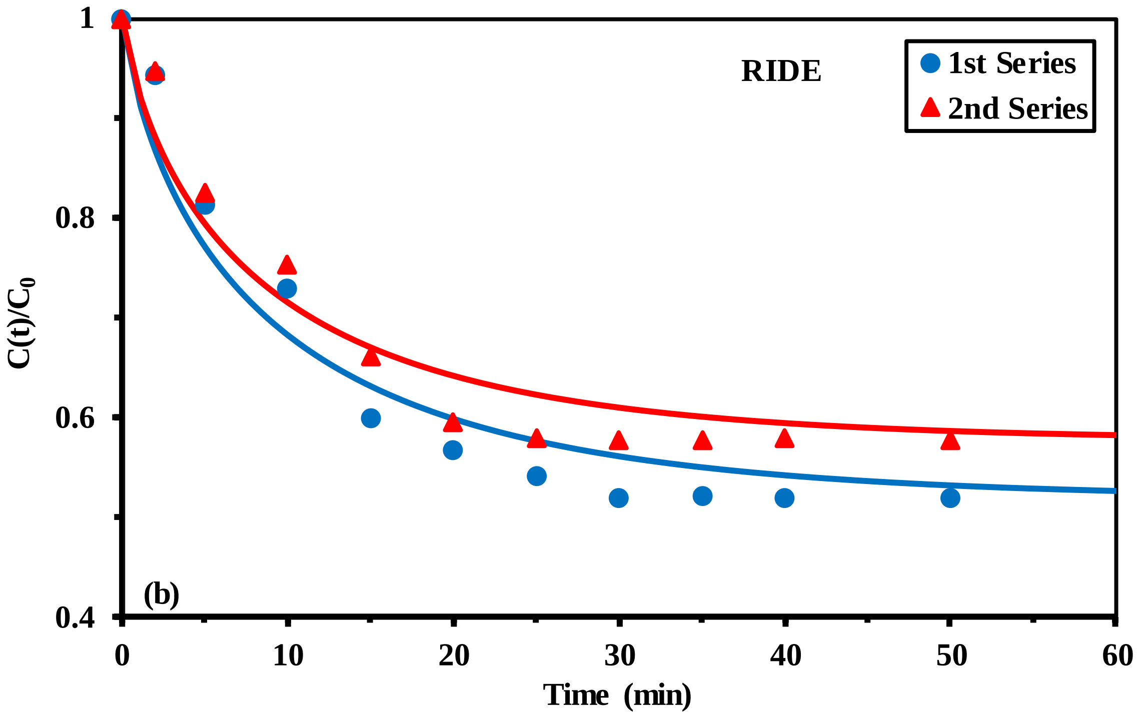

Figure S5. U(VI) uptake kinetics using Q-APEI sorbent – Modeling with the PSORE (a) and the RIDE (b) (SD: 0.3 g L^{-1}; pH_0: 4; pH_{eq}: 3.79-3.71; C_0: 0.214 mmol U L^{-1} and 0.249 mmol U L^{-1} for 1st and 2nd series, respectively; agitation speed: 170 rpm; T: 22 ± 2 °C).

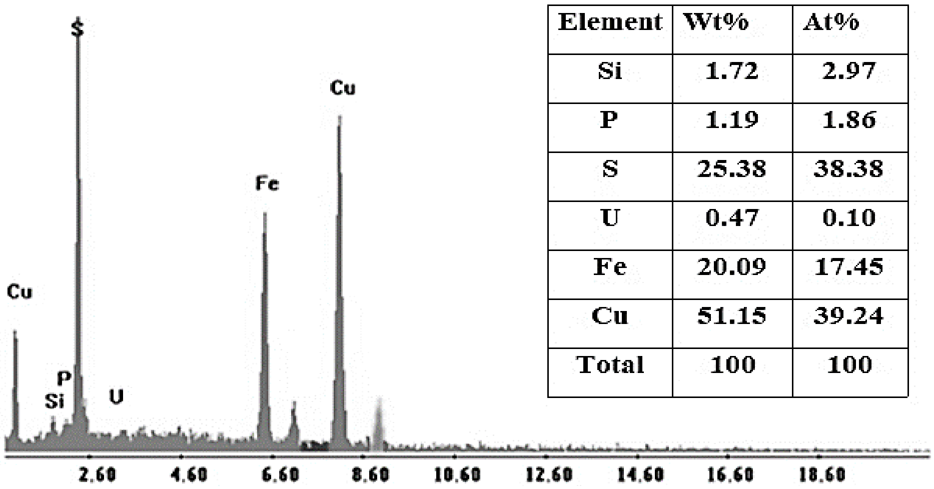

Element	Wt%	At%
Si	1.72	2.97
P	1.19	1.86
S	25.38	38.38
U	0.47	0.10
Fe	20.09	17.45
Cu	51.15	39.24
Total	100	100

Figure S6. Semi-quantitative EDX analysis of copper-cake collected after cementation.

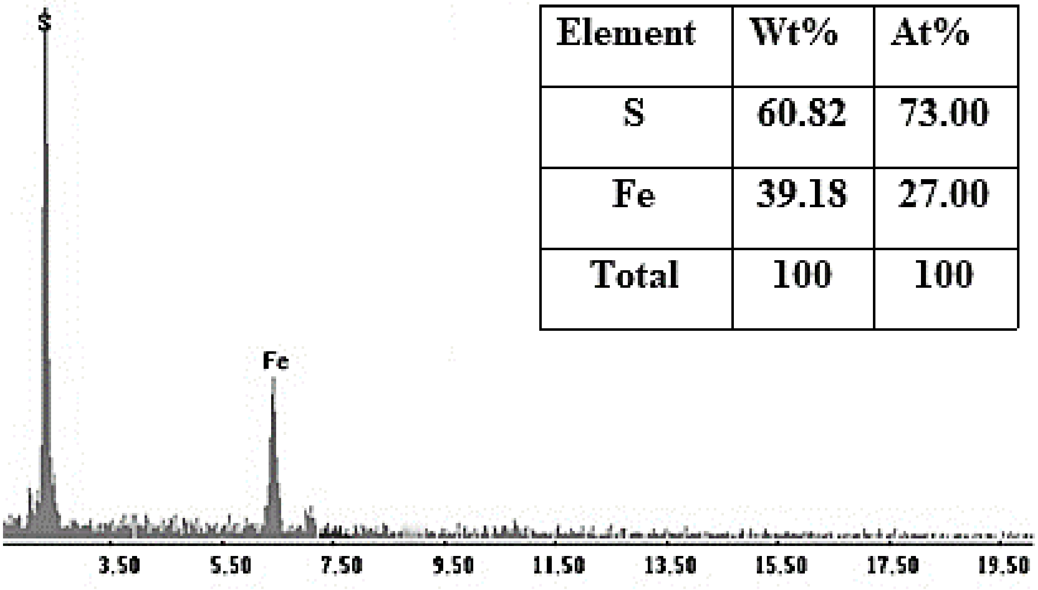

Element	Wt%	At%
S	60.82	73.00
Fe	39.18	27.00
Total	100	100

Figure S7. Semi-quantitative EDX analysis of iron-cake after pH control at pH 5.

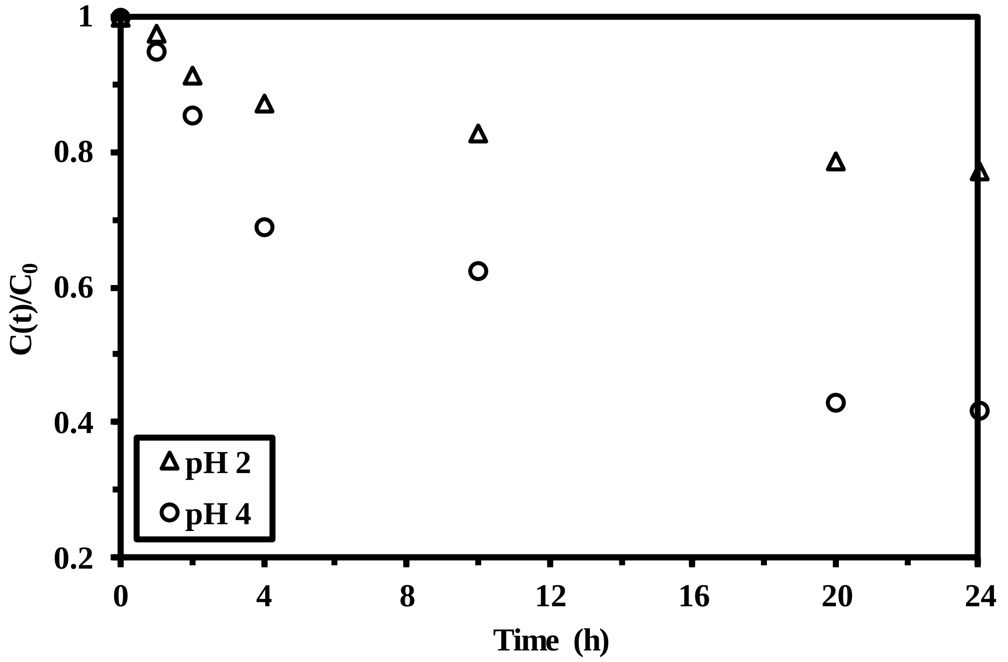

Figure S8. Effect of time on uranium sorption from multi-component solutions (C_0: 350-346 mg U L^{-1} = 1.47-1.45 mmol U L^{-1}; T: 22 ± 2 °C; SD: 0.125 g L^{-1}; agitation speed: 170 rpm).

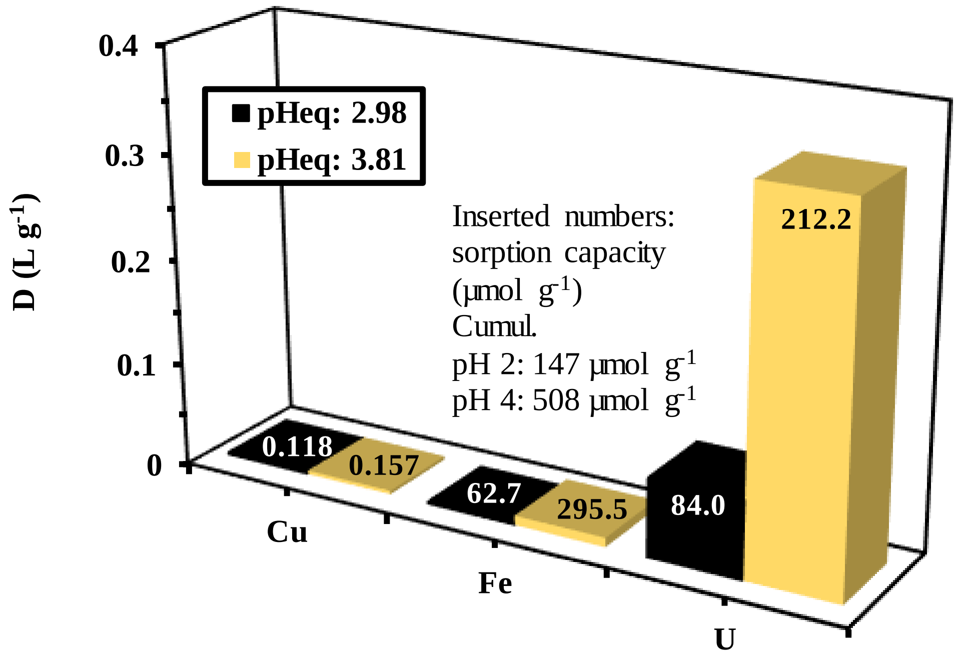

Figure S9. Distribution ratios of Cu, Fe and U after sorption on Q-APEI at pH_{eq}: 2.98 and 3.81 (pH_0: 2 and 4, respectively) from pre-treated PLS (inserted numbers represent the relevant sorption capacities at equilibrium, µmol g^{-1}).

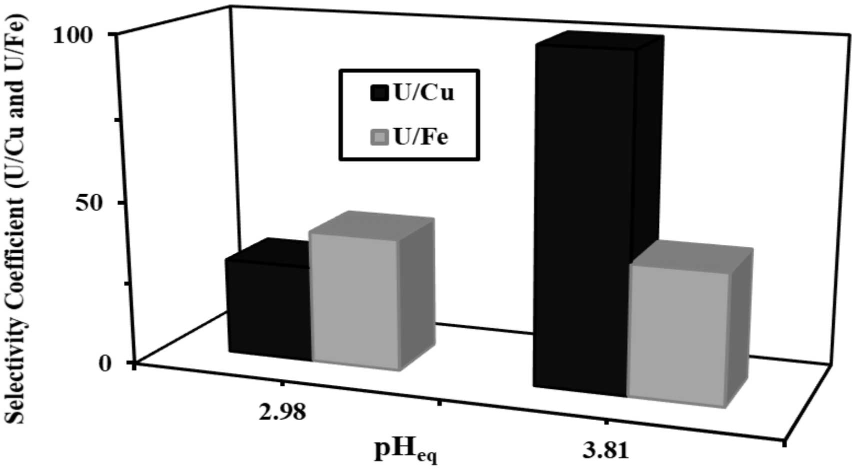

Figure S10. Selectively coefficients SC$_{U/Cu}$ and SC$_{U/Fe}$ for metal sorption from pre-treated PLS (C_0: 3 mg Cu L^{-1} = 0.047 mmol Cu L^{-1}, 1925 mg Fe L^{-1} = 34.47 mmol Fe L^{-1}, 350-346 mg U L^{-1} = 1.47-1.45 mmol U L^{-1}; SD: 0.125 g L^{-1}; agitation speed: 170 rpm; agitation time: 24 h).

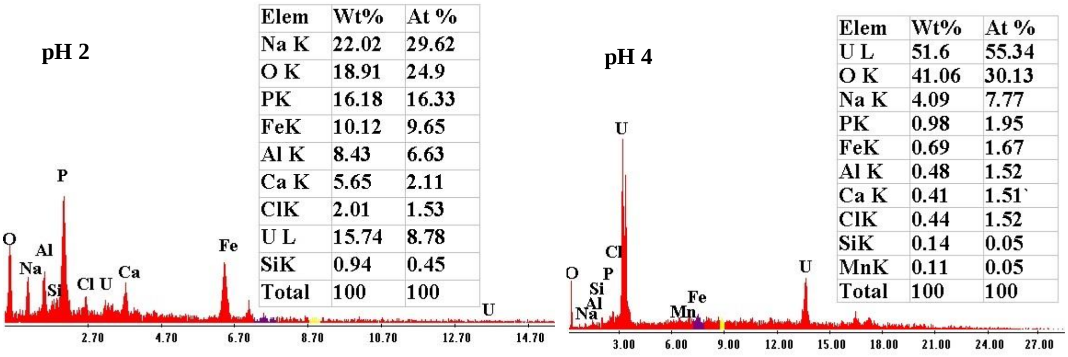

pH 2

Elem	Wt%	At %
Na K	22.02	29.62
O K	18.91	24.9
PK	16.18	16.33
FeK	10.12	9.65
Al K	8.43	6.63
Ca K	5.65	2.11
ClK	2.01	1.53
U L	15.74	8.78
SiK	0.94	0.45
Total	100	100

pH 4

Elem	Wt%	At %
U L	51.6	55.34
O K	41.06	30.13
Na K	4.09	7.77
PK	0.98	1.95
FeK	0.69	1.67
Al K	0.48	1.52
Ca K	0.41	1.51`
ClK	0.44	1.52
SiK	0.14	0.05
MnK	0.11	0.05
Total	100	100

Figure S11. Semi-quantitative analysis of the yellow cake produced from the eluates of sorbent after U-loading from ore leachates at pH 2 and pH 4.

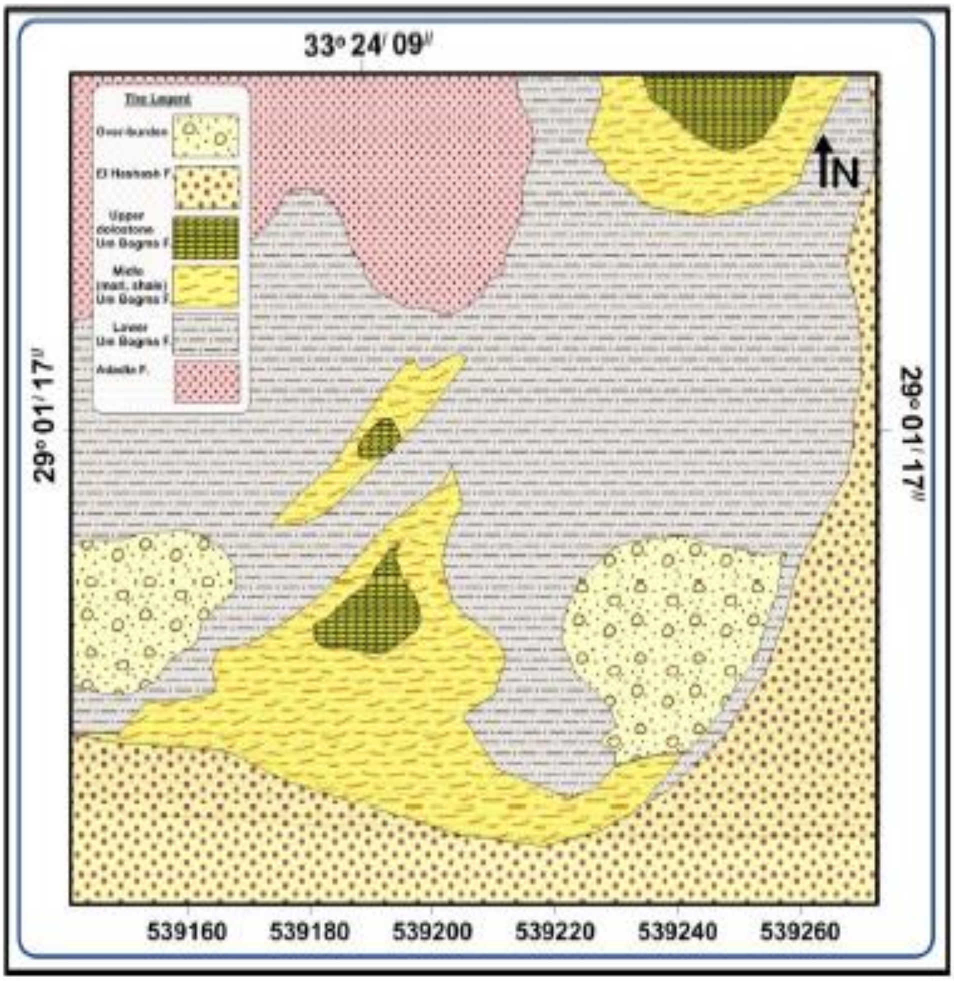

Figure S1. Alloga locality of Abu Zienema area, South Western Sinai, Egypt.

References

1. Hamza, M. F.; Aly, M. M.; Abdel-Rahman, A. A. H.; Ramadan, S.; Raslan, H.; Wang, S.; Vincent, T.; Guibal, E., Functionalization of magnetic chitosan particles for the sorption of U(VI), Cu(II) and Zn(II)—Hydrazide derivative of glycine-grafted chitosan. *Materials* **2017,** 10, (5), 539-560.
2. Hamza, M. F.; Abdel-Rahman, A. A. H., Extraction studies of some hazardous metal ions using magnetic peptide resins. *J. Dispersion Sci. Technol.* **2015,** 36, (3), 411-422.
3. Coates, J., Interpretation of Infrared Spectra, A Practical Approach. In *Encyclopedia of Analytical Chemistry*, Meyers, R. A., Ed. John Wiley & Sons Ltd: Chichester, U.K., 2000; pp 10815-10837.
4. Mohammadi, N.; Ganesan, A.; Chantler, C. T.; Wang, F., Differentiation of ferrocene D-5d and D-5h conformers using IR spectroscopy. *J. Organomet. Chem.* **2012,** 713, 51-59.
5. Colthup, N. B.; Daly, L. H.; Wiberley, S. E., *Introduction to Infrared and Raman Spectroscopy*. 3rd. ed. ed.; Academic Press, Inc.: San Diego, CA (USA), 1990; p 560.
6. Hu, X. J.; Wang, J. S.; Liu, Y. G.; Li, X.; Zeng, G. M.; Bao, Z. L.; Zeng, X. X.; Chen, A. W.; Long, F., Adsorption of chromium (VI) by ethylenediamine-modified cross-linked magnetic chitosan resin: Isotherms, kinetics and thermodynamics. *J. Hazard. Mater.* **2011,** 185, (1), 306-314.

7. Chen, Y.; Geng, J.; Zhuang, Y.; Zhao, J.; Chu, L.; Luo, X.; Zhao, Y.; Guo, Y., Preparation of the chitosan grafted poly (quaternary ammonium)/Fe_3O_4 nanoparticles and its adsorption performance for food yellow 3. *Carbohydr. Polym.* **2016,** 152, 327-336.
8. Hosoba, M.; Oshita, K.; Katarina, R. K.; Takayanagi, T.; Oshima, M.; Motomizu, S., Synthesis of novel chitosan resin possessing histidine moiety and its application to the determination of trace silver by ICP-AES coupled with triplet automated-pretreatment system. *Anal. Chim. Acta* **2009,** 639, (1-2), 51-56.
9. Oshita, K.; Takayanagi, T.; Oshima, M.; Motomizu, S., Adsorption behavior of cationic and anionic species on chitosan resins possessing amino acid moieties. *Anal. Sci.* **2007,** 23, (12), 1431-1434.
10. Cao, P. G.; Yao, J. L.; Bin, R.; Gu, R. N.; Tian, Z. Q., Surface-enhanced Raman scattering spectra of thiourea adsorbed at an iron electrode in $NaClO_4$ solution. *J. Phys. Chem. B* **2002,** 106, (39), 10150-10156.
11. Ho, Y. S.; McKay, G., Pseudo-second order model for sorption processes. *Process Biochem.* **1999,** 34, (5), 451-465.
12. Crank, J., *The Mathematics of Diffusion*. 2nd. ed.; Oxford University Press: Oxford, U.K., 1975; p 414.
13. Tien, C., *Adsorption Calculations and Modeling*. Butterworth-Heinemann: Newton, MA, 1994; p 243.
14. Foo, K. Y.; Hameed, B. H., Insights into the modeling of adsorption isotherm systems. *Chem. Eng. J.* **2010,** 156, (1), 2-10.
15. Gustafsson, J. P. *Visual MINTEQ,*, ver. 3.1; KTH, Royal Institute of Technology: Stockholm, Sweden, 2013.

© 2020 by the authors. Submitted for possible open access publication under the terms and conditions of the Creative Commons Attribution (CC BY) license (http://creativecommons.org/licenses/by/4.0/).
